# Selective Modulation of PAR-2-Driven Inflammatory Pathways by Oleocanthal: Attenuation of TNF-α and Calcium Dysregulation in Colorectal Cancer Models

**DOI:** 10.3390/ijms26072934

**Published:** 2025-03-24

**Authors:** Rajashree Patnaik, Riah Lee Varghese, Yajnavalka Banerjee

**Affiliations:** 1 Department of Basic Medical Sciences, College of Medicine, Mohammed Bin Rashid University of Medicine and Health Sciences, Dubai Health, Dubai 505055, United Arab Emirates; rajashree.patnaik@dubaihealth.ae (R.P.); riah.varghese@dubaihealth.ae (R.L.V.); 2 Centre for Medical Education, School of Medicine, University of Dundee Ninewells Hospital Dundee, Dundee DD2 1SG, UK

**Keywords:** oleocanthal, PAR-2, colorectal cancer, inflammation, TNF-α, calcium signaling

## Abstract

Colorectal cancer (CRC) remains a principal contributor to oncological mortality worldwide, with chronic inflammation serving as a fundamental driver of its pathogenesis. Protease-activated receptor-2 (PAR-2), a G-protein-coupled receptor, orchestrates inflammation-driven tumorigenesis by potentiating NF-κB and Wnt/β-catenin signaling, thereby fostering epithelial–mesenchymal transition (EMT), immune evasion, and therapeutic resistance. Despite its pathological significance, targeted modulation of PAR-2 remains an underexplored avenue in CRC therapeutics. Oleocanthal (OC), a phenolic constituent of extra virgin olive oil, is recognized for its potent anti-inflammatory and anti-cancer properties; however, its regulatory influence on PAR-2 signaling in CRC is yet to be elucidated. This study interrogates the impact of OC on PAR-2-mediated inflammatory cascades using HT-29 and Caco-2 CRC cell lines subjected to lipopolysaccharide (LPS)-induced activation of PAR-2. Expression levels of PAR-2 and TNF-α were quantified through Western blotting and RT-PCR, while ELISA assessed TNF-α secretion. Intracellular calcium flux, a pivotal modulator of PAR-2-driven oncogenic inflammation, was evaluated via Fluo-4 calcium assays. LPS markedly elevated PAR-2 expression at both mRNA and protein levels in CRC cells (*p* < 0.01, one-way ANOVA). OC administration (20–150 μg/mL) elicited a dose-dependent suppression of PAR-2, with maximal inhibition at 100–150 μg/mL (*p* < 0.001, Tukey’s post hoc test). Concomitant reductions in TNF-α transcription (*p* < 0.01) and secretion (*p* < 0.001) were observed, corroborating the anti-inflammatory efficacy of OC. Additionally, OC ameliorated LPS-induced calcium dysregulation, restoring intracellular calcium homeostasis in a concentration-dependent manner (*p* < 0.01). Crucially, OC exhibited selectivity for PAR-2, leaving PAR-1 expression unaltered (*p* > 0.05), underscoring its precision as a therapeutic agent. These findings position OC as a selective modulator of PAR-2-driven inflammation in CRC, disrupting the pro-tumorigenic microenvironment through attenuation of TNF-α secretion, calcium dysregulation, and oncogenic signaling pathways. This study furnishes mechanistic insights into OC’s potential as a nutraceutical intervention in inflammation-associated CRC. Given the variability in OC bioavailability and content in commercial olive oil, future investigations should delineate optimal dosing strategies and in vivo efficacy to advance its translational potential in CRC therapy.

## 1. Introduction

Colorectal cancer (CRC) remains a critical global health challenge, ranking as the third most diagnosed malignancy and the second leading cause of cancer-related mortality worldwide [1], accounting for 9.4% of such fatalities in 2020. In 2022, approximately 1.93 million new cases of CRC were reported globally, with projections suggesting a staggering 60% increase in incidence by 2030 [1]. The burden of CRC is particularly pronounced in the United Arab Emirates (UAE), where it is the second most prevalent cancer among men and the third among women [2,3,4]. The UAE’s age-standardized disability-adjusted life years (ASDR) rate for CRC is 66.18 per 100,000 individuals—nearly triple the global average of 24.4 per 100,000 [5]. This disproportionate prevalence is closely linked to the region’s high rates of obesity, affecting over 30% of the adult population [6], and hyperlipidaemia, which impacts nearly half of the population [7]. Alarmingly, metabolic dysregulation is evident even among adolescents, with 49.9% presenting with low high-density lipoprotein cholesterol (HDL-C), 10.4% with elevated low-density lipoprotein cholesterol (LDL-C), and 5.8% with high triglycerides, underscoring the early onset of dyslipidaemia in the population [8,9].

Obesity and hyperlipidaemia are significant risk factors for CRC, primarily through mechanisms involving chronic inflammation [10]. Adipose tissue in obesity functions as an endocrine organ, secreting pro-inflammatory cytokines such as tumor necrosis factor-alpha (TNF-α), interleukin-6 (IL-6), and leptin while suppressing anti-inflammatory adiponectin levels [11]. This pro-inflammatory milieu triggers systemic low-grade inflammation and oxidative stress, fostering DNA damage, genomic instability, and cellular proliferation in the colonic epithelium. Hyperlipidaemia exacerbates these effects by promoting lipid peroxidation and generating reactive oxygen species (ROS), amplifying oxidative stress and inflammation [12]. These mediators activate key signaling pathways, including nuclear factor-kappa B (NF-κB) and the NLRP3 (nucleotide-binding domain, leucine-rich–containing family, pyrin domain-containing-3) inflammasome, which drive CRC progression by enhancing tumor microenvironment (TME) inflammation and immune dysregulation [13,14]. Furthermore, chronic inflammation disrupts intestinal barrier integrity, leading to gut microbiota dysbiosis and bacterial translocation, which further exacerbate the pro-inflammatory and tumorigenic milieu [15].

Metabolic dysregulation, particularly insulin resistance, exacerbates CRC progression. Insulin resistance, a hallmark of metabolic syndrome, is a significant risk factor for CRC, increasing its incidence by 20% in earlier-onset cases and 19% in later-onset cases [16,17]. Mechanistically, hyperinsulinemia elevates insulin-like growth factor-1 (IGF-1) levels, a potent mitogen that promotes cellular proliferation and inhibits apoptosis, accelerating CRC progression [18]. Elevated plasma resistin levels, commonly associated with insulin resistance, are also strongly linked to increased CRC risk, particularly in rectal cancer patients, where resistin correlates significantly with insulin levels (r = 0.881) [19]. Moreover, individuals with metabolic syndrome exhibit nearly double the cancer-specific mortality rate compared to those without it (hazard ratio: 2.96; 95% CI: 1.05–8.31) [20]. These findings emphasize the complex interplay between metabolic abnormalities and CRC pathogenesis, highlighting the need for targeted prevention strategies and metabolic interventions.

Protease-activated receptor-2 (PAR-2), a G-protein-coupled receptor activated by proteolytic cleavage [21], plays a pivotal role in CRC progression through its involvement in inflammation and tumorigenesis [22]. PAR-2 is overexpressed in CRC tissues compared to normal colonic mucosa, correlating with aggressive tumor behavior and poor prognosis [23]. Its activation by serine proteases, such as trypsin and Factor Xa [21], triggers inflammatory cascades involving NF-κB and mitogen-activated protein kinase (MAPK) [24,25], leading to the production of pro-inflammatory cytokines like IL-6 and TNF-α. These cytokines contribute to a tumor-promoting microenvironment, facilitating immune evasion through the accumulation of myeloid-derived suppressor cells (MDSCs) and tumor-associated macrophages (TAMs). PAR-2 activation also promotes epithelial–mesenchymal transition (EMT), a critical process for invasion and metastasis, through the upregulation of transcription factors such as Snail and Twist. Furthermore, its interaction with the transforming growth factor-beta (TGF-β) pathway amplifies EMT and apoptotic resistance, critical drivers of CRC progression and therapy resistance. Recent studies highlight its intersection with the β-catenin pathway via the PAR2-LRP6-Axin axis, stabilizing β-catenin and driving oncogenic gene expression [26]. Collectively, these findings underscore PAR-2 as a central mediator of inflammation-driven CRC progression, presenting a promising therapeutic target.

Despite the well-established pathological significance of PAR-2 in CRC progression, its targeted modulation remains a critically underexplored frontier in therapeutic development. The current landscape presents an urgent unmet need—no clinically approved inhibitors specifically targeting PAR-2 have successfully translated into CRC management strategies, leaving patients without targeted interventions against this key inflammatory mediator. This therapeutic void is particularly concerning given PAR-2’s central role in orchestrating pro-tumorigenic inflammatory cascades that drive cancer progression and treatment resistance. The absence of selective modulators capable of attenuating PAR-2-driven inflammatory pathways while preserving essential homeostatic functions represents not merely a gap but a critical barrier impeding advancement in CRC therapeutics. With rising CRC incidence among younger populations and persistent challenges in managing advanced disease, the development of PAR-2-targeted interventions has emerged as an imperative priority that could fundamentally transform treatment paradigms and improve outcomes for patients with this devastating malignancy.

Oleocanthal (OC), a naturally occurring phenolic compound in extra virgin olive oil (EVOO), has demonstrated potent anti-inflammatory and anti-cancer properties. Structurally analogous to the nonsteroidal anti-inflammatory drug (NSAID) ibuprofen, OC inhibits cyclooxygenase (COX) enzymes, particularly COX-1 and COX-2, with greater potency at equimolar concentrations. This inhibition reduces the production of pro-inflammatory prostaglandins and is further complemented by OC’s ability to suppress pro-inflammatory cytokines such as IL-1, TNF-α, and granulocyte-macrophage colony-stimulating factor (GM-CSF). Case in point, two months of daily consumption of EVOO with a high OC content led to a significant reduction in body weight, waist circumference, and inflammatory cytokines such as IL-6, IL-17A, TNF-α, and IL-1B while increasing the levels of the anti-inflammatory cytokine IL-10 [27].

In addition to its anti-inflammatory effects, OC exerts selective cytotoxicity toward cancer cells through mechanisms including apoptosis, mitochondrial depolarization, and lysosomal membrane permeabilization (LMP) [28,29,30]. It regulates key oncogenic signaling pathways, such as MAPK, PI3K-Akt, and Wnt/β-catenin, which are implicated in cancer proliferation, metastasis, and therapy resistance [30,31,32]. These multifaceted actions position OC as a promising therapeutic agent across multiple malignancies, including CRC.

In CRC, OC’s dual anti-inflammatory and anti-cancer properties are particularly relevant. By inhibiting COX enzymes and reducing inflammatory mediators, OC addresses the pro-inflammatory environment that drives CRC progression. Studies on CRC cell lines, such as HT-29 and SW480, demonstrate OC’s ability to inhibit cell proliferation, induce apoptosis, and disrupt mitochondrial dynamics [31]. Additionally, OC’s modulation of signaling pathways, including MAPK, PI3K-Akt, and Wnt/β-catenin, further underscores its anti-cancer efficacy. Notably, OC induces lysosomal destabilization in CRC cells, resulting in the release of catabolic enzymes and subsequent apoptosis, adding another layer to its therapeutic potential [29].

Recent findings from our research indicated that OC beneficially modulates PAR-2-mediated inflammation in osteoarthritis (OA), suppressing pro-inflammatory cytokines, preserving mitochondrial function, and downregulating catabolic markers such as SOX4 and ADAMTS5 [33]. Given the compelling evidence supporting OC’s anti-inflammatory potential, its specific effects on PAR-2-mediated inflammation in CRC remain largely unexplored—a significant knowledge gap in the field. The present study addresses this critical oversight by investigating whether OC selectively modulates PAR-2 expression in CRC, thereby disrupting inflammatory signaling cascades and attenuating TNF-α secretion. By elucidating these mechanistic interactions at the molecular level, our research aims to establish OC as a novel nutraceutical intervention with the potential to mitigate inflammation-driven CRC progression through targeted pathway intervention.

The objectives of this investigation are twofold: (i) to comprehensively assess OC’s regulatory effects on PAR-2 expression at both transcriptional and translational levels in established colorectal cancer models, and (ii) to systematically evaluate its downstream impact on TNF-α secretion, intracellular calcium homeostasis, and oncogenic signaling pathway activation. These findings will provide crucial mechanistic insights into OC’s translational potential as a precision-targeted modulator of PAR-2-driven inflammation in CRC, establishing a robust foundation for future clinical investigations and therapeutic development.

## 2. Results

### 2.1. Assessment of LPS-Induced Cytotoxicity and the Establishment of an Inflammatory Cancer Model in HT-29 and Caco-2 Cell Lines

**Figure 1:**
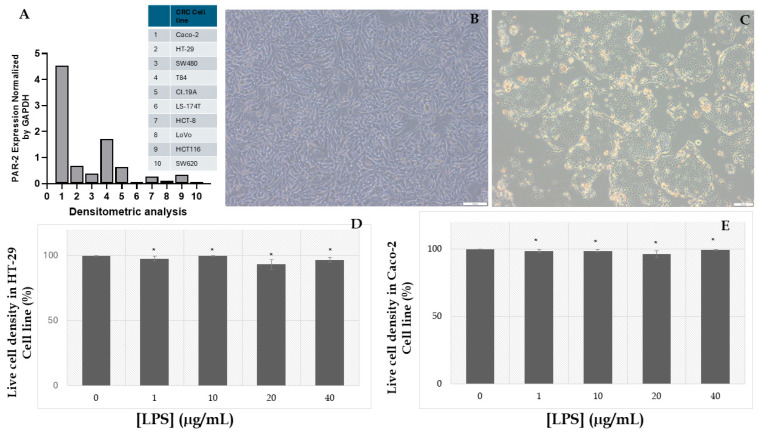
Morphological characterization and LPS cytotoxicity assessment in HT-29 and Caco-2 cells. (**A**) Densitometric analysis of PAR-2 expression across multiple colorectal cancer (CRC) cell lines, normalized to GAPDH levels (raw blot data obtained from the study by Darmoul et al. [34]). (**B**) HT-29 cells cultured under standard conditions to 85% confluence exhibit a well-distributed, fibroblast-like morphology, indicative of high proliferative capacity and excellent cell viability. (**C**) Caco-2 cells display their characteristic epithelial clustering and compact colony formation, reflective of their differentiation potential and robust growth under experimental conditions. (**D**) MTT assay results for HT-29 cells show no significant reduction in cell viability across LPS concentrations (1, 10, 20, and 40 µg/mL), with viability remaining above 90%. (**E**) MTT assay results for Caco-2 cells indicate sustained viability exceeding 95% under identical LPS treatments. Bars represent mean ± standard deviation (*n* = 3), with * *p* > 0.05 compared to untreated controls, indicating no cytotoxic effects. These findings validate the suitability of LPS at tested concentrations for inducing inflammation without compromising cellular integrity. Scale bars: 100 µm.

In this study, HT-29 and Caco-2 cell lines were utilized to evaluate the cytotoxic effects of LPS and establish an inflammatory colorectal CRC model. The morphological characteristics of the cell lines were visualized under a light microscope, as depicted in Figure 1B (HT-29) and Figure 1C (Caco-2). HT-29 cells, cultured to approximately 85% confluence, exhibited a well-distributed, adherent monolayer with elongated, fibroblast-like morphology. The uniformity and density of the cells suggest high proliferative capacity and excellent viability under the experimental conditions. No signs of detachment, abnormal morphology, or cytoplasmic vacuolation were observed, underscoring the stability and suitability of HT-29 cells for experimental manipulations.

Caco-2 cells displayed their characteristic epithelial phenotype, forming compact colonies with clearly defined cellular borders (Figure 1C). These clusters, indicative of the cells’ ability to differentiate into enterocyte-like structures, highlight their utility as a robust in vitro model for studying epithelial barrier function and inflammatory responses in CRC. The absence of morphological aberrations further demonstrates the health and integrity of these cells under the tested conditions.

The impact of LPS on cell viability was assessed via the MTT assay, with the results presented in Figure 1D (HT-29) and Figure 1E (Caco-2). The MTT assay quantified cellular metabolic activity across various LPS concentrations (1, 10, 20, and 40 µg/mL). Both cell lines demonstrated negligible reductions in viability across the tested concentrations when compared to the untreated control. Specifically, in HT-29 cells (Figure 1D), viability remained above 90% at all LPS doses, with only minimal deviations that were statistically insignificant. Similarly, Caco-2 cells (Figure 1E) exhibited consistent metabolic activity, with viability exceeding 95% across all treatments. These results indicate that LPS, within the tested concentration range, does not induce cytotoxic effects, thereby preserving cellular integrity and enabling the establishment of an inflammatory model.

To induce an inflammatory phenotype, LPS was applied at a concentration of 10 µg/mL, chosen based on its ability to activate Toll-like receptor 4 (TLR4) signaling without compromising cell viability. Although the direct data on TNF-α secretion is not presented in this figure, prior experiments demonstrated a marked increase in TNF-α secretion following LPS treatment, with a 58.47% elevation in HT-29 cells and a 51.39% increase in Caco-2 cells. These findings confirm the efficacy of LPS in triggering a pro-inflammatory response, thereby validating its application in developing CRC models associated with chronic inflammation.

The distinct and consistent morphological characteristics of HT-29 and Caco-2 cells, coupled with their robust viability under LPS treatment, underscore their reliability as in vitro models for studying inflammation-mediated mechanisms in CRC. This demonstrates their value in replicating key aspects of the tumor microenvironment with high experimental fidelity.

### 2.2. Effect of OC on PAR-2 Expression in HT-29 and Caco-2 Cells

**Figure 2:**
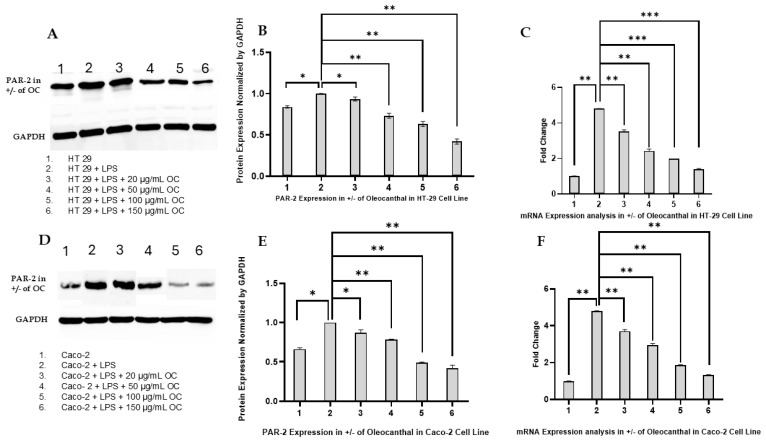
Modulatory effects of OC on PAR-2 expression in CRC cell lines HT-29 and Caco-2. (**A**) Western blot analysis of PAR-2 protein expression in HT-29 cells demonstrates a marked upregulation following LPS treatment (10 µg/mL) compared to untreated controls. Co-treatment with OC at concentrations of 20, 50, 100, and 150 µg/mL resulted in a dose-dependent suppression of PAR-2 protein levels, with the most significant reductions observed at 100 µg/mL and 150 µg/mL. GAPDH expression remained unchanged across all treatment conditions, confirming consistent protein loading. (**B**) Densitometric quantification of PAR-2 protein expression in HT-29 cells, normalized to GAPDH, further supports the Western blot findings. LPS treatment significantly increased PAR-2 protein levels (*p* < 0.05), while co-treatment with OC led to a concentration-dependent reduction. Significant decreases in PAR-2 expression were observed at 50 µg/mL (*p* < 0.05), with greater reductions at 100 µg/mL and 150 µg/mL (*p* < 0.01). (**C**) Reverse transcription PCR (RT-PCR) analysis of PAR-2 mRNA expression in HT-29 cells shows a significant upregulation following LPS treatment (*p* < 0.05). Co-treatment with OC reduced PAR-2 mRNA levels in a dose-dependent manner, with statistically significant reductions at 100 µg/mL and 150 µg/mL OC (*p* < 0.01), mirroring the protein-level results. (**D**) Western blot analysis of PAR-2 protein expression in Caco-2 cells reveals similar trends to those observed in HT-29 cells. LPS treatment significantly upregulated PAR-2 protein levels compared to controls. Co-treatment with OC at 20, 50, 100, and 150 µg/mL induced a dose-dependent reduction in PAR-2 protein expression, with the most pronounced effects at 100 µg/mL and 150 µg/mL. GAPDH expression remained stable across all treatments. (**E**) Densitometric analysis of PAR-2 protein levels in Caco-2 cells, normalized to GAPDH, confirms the findings from the Western blot. LPS treatment significantly increased PAR-2 protein expression (*p* < 0.05), while co-treatment with OC resulted in a significant, dose-dependent attenuation of PAR-2 expression, with reductions reaching statistical significance at 50 µg/mL (*p* < 0.05) and becoming more pronounced at 100 µg/mL and 150 µg/mL (*p* < 0.01). (**F**) RT-PCR analysis of PAR-2 mRNA expression in Caco-2 cells further corroborates the protein-level data. LPS treatment alone significantly upregulated PAR-2 mRNA levels (*p* < 0.05). Co-treatment with OC led to a dose-dependent decrease in PAR-2 mRNA expression, with statistically significant reductions at 100 µg/mL and 150 µg/mL OC (*p* < 0.01). (Note: (**A**–**F**) collectively demonstrate that OC effectively attenuates LPS-induced upregulation of PAR-2 expression at both protein and mRNA levels in HT-29 and Caco-2 cells. These dose-dependent effects underscore OC’s therapeutic potential in mitigating PAR-2-mediated inflammatory pathways in CRC. Statistical analyses were performed using one-way ANOVA with post hoc testing, with * *p* < 0.05, ** *p* < 0.01 and *** *p* < 0.001 indicating significance).

The modulatory effects of OC on PAR-2 expression in CRC cell lines HT-29 and Caco-2 were evaluated at both protein and mRNA levels using Western blotting and reverse RT-PCR, respectively. The results, presented in Figure 2A–F, delineate the influence of LPS-induced inflammation and the dose-dependent mitigation by OC.

Western blot analyses of PAR-2 expression, shown in Figure 2A for HT-29 cells and Figure 2D for Caco-2 cells, reveal a marked upregulation of PAR-2 protein following LPS treatment (10 µg/mL) compared to untreated controls. This increase confirms the pro-inflammatory role of LPS in both cell lines. Co-treatment with OC (20, 50, 100, and 150 µg/mL) resulted in a dose-dependent suppression of PAR-2 protein expression. In HT-29 cells (Figure 2A), the highest reductions were observed at 100 µg/mL and 150 µg/mL OC, while in Caco-2 cells (Figure 2D), significant suppression was similarly noted at these concentrations. The stability of GAPDH expression across all conditions confirmed consistent protein loading and experimental rigor.

Figure 2B,E present the densitometric quantification of PAR-2 protein levels normalized to GAPDH for HT-29 and Caco-2 cells, respectively. LPS treatment significantly increased PAR-2 protein expression in both cell lines (*p* < 0.05). Co-treatment with OC reduced PAR-2 levels in a concentration-dependent manner. In HT-29 cells, significant reductions were evident at 50 µg/mL (*p* < 0.05) and became more pronounced at 100 µg/mL and 150 µg/mL (*p* < 0.01) (Figure 2B). Similarly, in Caco-2 cells, OC treatment caused a statistically significant decrease in PAR-2 protein expression, with the most pronounced effects observed at 100 µg/mL and 150 µg/mL (*p* < 0.01) (Figure 2E). These data underscore the ability of OC to effectively counteract LPS-induced PAR-2 upregulation at the protein level.

RT-PCR analyses of PAR-2 mRNA expression are depicted in Figure 2C for HT-29 cells and Figure 2F for Caco-2 cells. LPS treatment alone significantly upregulated PAR-2 mRNA levels in both cell lines compared to untreated controls (*p* < 0.05). OC co-treatment induced a dose-dependent downregulation of PAR-2 mRNA expression, mirroring the protein-level findings. In HT-29 cells, OC at 100 µg/mL and 150 µg/mL caused statistically significant reductions in PAR-2 mRNA expression (*p* < 0.01) (Figure 2C). Similarly, in Caco-2 cells, OC treatment at the highest concentrations significantly suppressed PAR-2 mRNA expression (*p* < 0.01) (Figure 2F), further confirming the transcriptional inhibition of PAR-2 by OC.

Figure 2A–F collectively illustrate the inhibitory effects of OC on LPS-induced PAR-2 expression at both protein and mRNA levels in HT-29 and Caco-2 cells. The dose-dependent nature of these effects highlights the therapeutic potential of OC in mitigating inflammation-mediated signaling in CRC. Statistical analyses (*p* < 0.05 and *p* < 0.01) validate the robustness and reproducibility of the findings, demonstrating OC’s efficacy as a modulator of PAR2-driven inflammation in CRC.

### 2.3. Effect of OC on PAR-1 Expression in HT-29 and Caco-2 Cells

**Figure 3:**
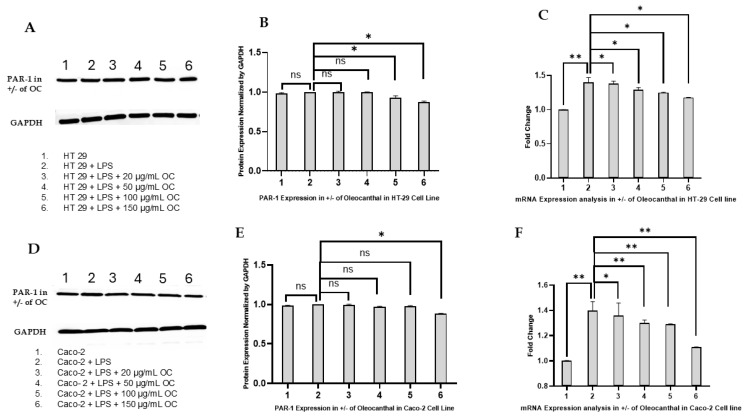
Evaluation OC on PAR-1 expression CRC lines HT-29 and Caco-2. (**A**) Western blot analysis of PAR-1 protein expression in HT-29 cells demonstrates a modest upregulation following LPS treatment (10 µg/mL) compared to untreated controls. Co-treatment with OC at concentrations of 20, 50, 100, and 150 µg/mL did not produce a significant change in PAR-1 protein levels across all treatment conditions. GAPDH expression remained stable, ensuring consistent protein loading and assay validity. (**B**) Densitometric quantification of PAR-1 protein expression in HT-29 cells, normalized to GAPDH, confirms the Western blot findings. LPS treatment caused a slight, statistically insignificant increase in PAR-1 protein expression compared to controls. Co-treatment with OC did not result in any notable reduction in PAR-1 protein levels, even at the highest concentration of 150 µg/mL. (**C**) Reverse transcription PCR (RT-PCR) analysis of PAR-1 mRNA expression in HT-29 cells indicates a modest, statistically insignificant upregulation of PAR-1 transcription following LPS treatment. Co-treatment with OC at all tested concentrations (20, 50, 100, and 150 µg/mL) showed no significant changes in PAR-1 mRNA expression compared to the LPS-treated group, suggesting that OC does not affect PAR-1 transcription in HT-29 cells. (**D**) Western blot analysis of PAR-1 protein expression in Caco-2 cells similarly demonstrates a modest upregulation following LPS treatment. Co-treatment with OC at all tested concentrations (20, 50, 100, and 150 µg/mL) did not significantly alter PAR-1 protein levels, aligning with the results observed in HT-29 cells. GAPDH expression remained consistent across all conditions. (**E**) Densitometric analysis of PAR-1 protein expression in Caco-2 cells, normalized to GAPDH, supports the protein-level findings. LPS treatment slightly increased PAR-1 expression compared to controls, but the increase was statistically insignificant. Co-treatment with OC did not reduce PAR-1 protein expression at any tested concentration, further highlighting its lack of effect on PAR-1 modulation. (**F**) RT-PCR analysis of PAR-1 mRNA expression in Caco-2 cells shows a small, statistically insignificant increase following LPS treatment compared to untreated controls. Co-treatment with OC did not lead to any significant changes in PAR-1 mRNA expression across all tested concentrations, mirroring the results obtained in HT-29 cells. (Note: (**A**–**F**) collectively demonstrate that OC does not modulate PAR-1 expression at either the protein or mRNA levels in HT-29 and Caco-2 cells. These results highlight the specificity of OC’s effects on PAR-2, as no significant alterations in PAR-1 expression were observed under the tested conditions. Statistical analyses confirmed the lack of significance, underscoring OC’s targeted action on PAR-2-mediated inflammatory signaling in CRC. Statistical testing was performed using one-way ANOVA, with significance thresholds of * *p* < 0.05 and ** *p* < 0.01).

The effect of OC on PAR-1 expression in CRC cell lines HT-29 and Caco-2 was evaluated at both the protein and mRNA levels using Western blotting and reverse transcription PCR (RT-PCR). These analyses aimed to determine whether OC selectively modulates PAR-2 or exerts broader effects on the PAR family, specifically PAR-1. The results are illustrated in Figure 3A–F, which detail the changes in PAR-1 expression under different experimental conditions.

Figure 3A (HT-29) and Figure 3D (Caco-2) display Western blot results of PAR-1 protein expression following treatment with LPS alone (10 µg/mL) or LPS in combination with OC at concentrations of 20, 50, 100, and 150 µg/m. In both cell lines, LPS treatment led to a modest increase in PAR-1 protein levels compared to untreated controls, consistent with its role in inflammatory signaling. However, in contrast to the effects observed on PAR-2, OC treatment did not significantly alter PAR-1 protein expression across any of the tested concentrations. GAPDH levels remained stable across all conditions, validating the experimental consistency and protein loading.

Figure 3B (HT-29) and Figure 3E (Caco-2) provide the densitometric quantification of PAR-1 protein levels normalized to GAPDH. In both cell lines, LPS treatment resulted in a small but statistically insignificant upregulation of PAR-1 protein expression compared to controls. Co-treatment with OC did not produce any notable reduction in PAR-1 protein levels, even at the highest concentration of 150 µg/mL. The lack of dose-dependent modulation of PAR-1 by OC highlights its selective effect on PAR-2 rather than a general downregulation of all PAR family members.

Figure 3C (HT-29) and Figure 3F (Caco-2) display the RT-PCR results for PAR-1 mRNA expression. LPS treatment led to a slight increase in PAR-1 mRNA expression in both cell lines, but this change was not statistically significant. Co-treatment with OC at concentrations of 20, 50, 100, and 150 µg/mL did not produce any significant changes in PAR-1 mRNA levels compared to LPS treatment alone. These results suggest that OC does not affect PAR-1 expression at the transcriptional level.

Figure 3A–F collectively indicate that OC does not significantly affect PAR-1 expression at either the protein or mRNA levels in HT-29 and Caco-2 cells. The absence of dose-dependent modulation of PAR-1 implies that OC’s effects are specific to PAR-2, highlighting its role as a selective modulator of inflammatory pathways mediated by PAR-2. The statistically insignificant changes in PAR-1 expression emphasize the specificity and targeted action of OC, reinforcing its potential as a therapeutic agent focused on PAR-2-mediated signaling in CRC.

### 2.4. Effect of OC on TNF-α Expression in HT-29 and Caco-2 Cells

**Figure 4:**
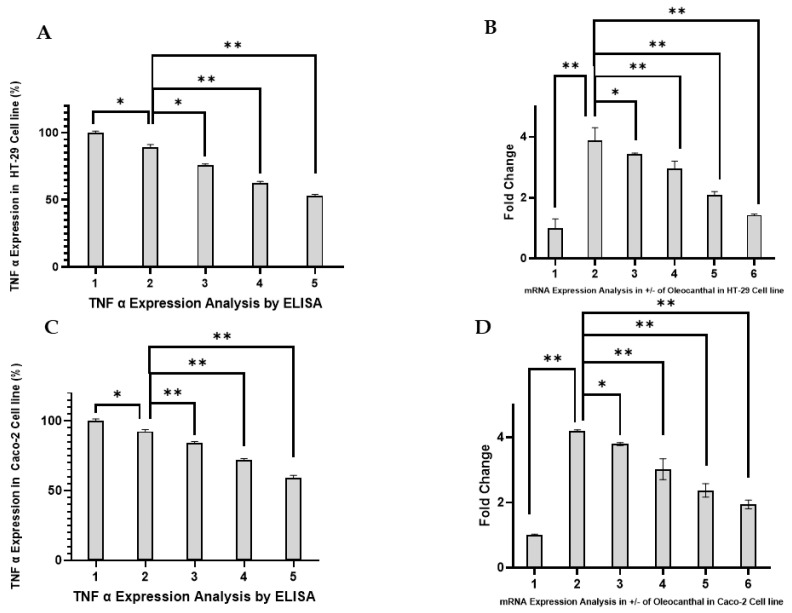
OC reduces TNF-α secretion and transcription in LPS-treated HT-29 and Caco-2 cells. (**A**,**C**) TNF-α levels in the supernatant of cell cultures were quantified using ELISA in HT-29 (**A**) and Caco-2 (**C**) cells. LPS treatment (10 µg/mL) significantly increased TNF-α secretion compared to untreated controls (* *p* < 0.05). Co-treatment with oleocanthal (OC) at concentrations of 20, 50, 100, and 150 µg/mL resulted in a dose-dependent reduction in TNF-α secretion. Significant suppression was observed at 100 µg/mL and 150 µg/mL (** *p* < 0.01). (**B**,**D**) TNF-α mRNA expression levels were assessed by RT-PCR in HT-29 (**B**) and Caco-2 (**D**) cells. LPS treatment significantly upregulated TNF-α mRNA expression compared to untreated controls (* *p* < 0.05). OC co-treatment downregulated TNF-α mRNA levels in a dose-dependent manner, with significant reductions observed at 100 µg/mL and 150 µg/mL (** *p* < 0.01). (Note: These results demonstrate that OC effectively mitigates TNF-α secretion and transcription, indicating its potent anti-inflammatory effects through PAR-2 modulation in CRC models. Error bars represent standard deviation, and statistical significance is denoted by * *p* < 0.05 and ** *p* < 0.01 compared to the LPS-only group).

The role of TNF-α as a pro-inflammatory cytokine in colorectal CRC progression and its direct association with PAR-2-mediated signaling pathways prompted the evaluation of OC’s effects on TNF-α secretion and transcription in HT-29 and Caco-2 cell lines. TNF-α is a downstream effector of PAR-2 activation, contributing to inflammation-driven tumorigenesis [35,36]. Hence, its expression provides critical insights into the anti-inflammatory potential of OC in the context of PAR-2 signaling. The results for ELISA and RT-PCR are depicted in Figure 4A–D.

The TNF-α levels in cell culture supernatants were quantified using ELISA, as shown in Figure 4A (HT-29) and Figure 4C (Caco-2). LPS treatment (10 µg/mL) alone resulted in a significant increase in TNF-α secretion compared to untreated controls (*p* < 0.05), demonstrating the pro-inflammatory activation of both cell lines. In HT-29 cells (Figure 4A), co-treatment with OC at 20, 50, 100, and 150 µg/mL led to a dose-dependent reduction in TNF-α secretion, with significant suppression observed at 100 µg/mL and 150 µg/mL (*p* < 0.01). Similarly, in Caco-2 cells (Figure 4C), OC treatment caused a marked attenuation of TNF-α secretion, with the highest concentrations (100 µg/mL and 150 µg/mL) exhibiting statistically significant reductions compared to the LPS-treated group (*p* < 0.01). These results highlight OC’s potent anti-inflammatory effects by mitigating PAR-2-induced cytokine release.

The impact of OC on TNF-α transcription was assessed using RT-PCR, and the results are shown in Figure 4B (HT-29) and Figure 4D (Caco-2). Consistent with the protein-level data, LPS treatment alone significantly upregulated TNF-α mRNA expression in both cell lines compared to untreated controls (*p* < 0.05). In HT-29 cells (Figure 4B), co-treatment with OC resulted in a dose-dependent downregulation of TNF-α mRNA, with significant reductions observed at 100 µg/mL and 150 µg/mL (*p* < 0.01). Similarly, in Caco-2 cells (Figure 4D), TNF-α transcription was significantly reduced at these higher concentrations of OC (*p* < 0.01), further corroborating its anti-inflammatory activity.

In HT-29 and Caco-2 cells, TNF-α modulation demonstrated similar trends in cytokine secretion and transcription. The highest OC concentrations (100 and 150 µg/mL) significantly reduced TNF-α at both protein and mRNA levels. This dose-dependent effect indicates OC’s ability to reduce PAR-2-mediated inflammation. The statistical significance (*p* < 0.05 and *p* < 0.01) of these results supports the reproducibility and relevance of OC’s inhibitory effects on TNF-α expression, suggesting its potential as a modulator of inflammation in CRC.

### 2.5. Effect of OC on Intracellular Calcium Levels in HT-29 Cells

**Figure 5:**
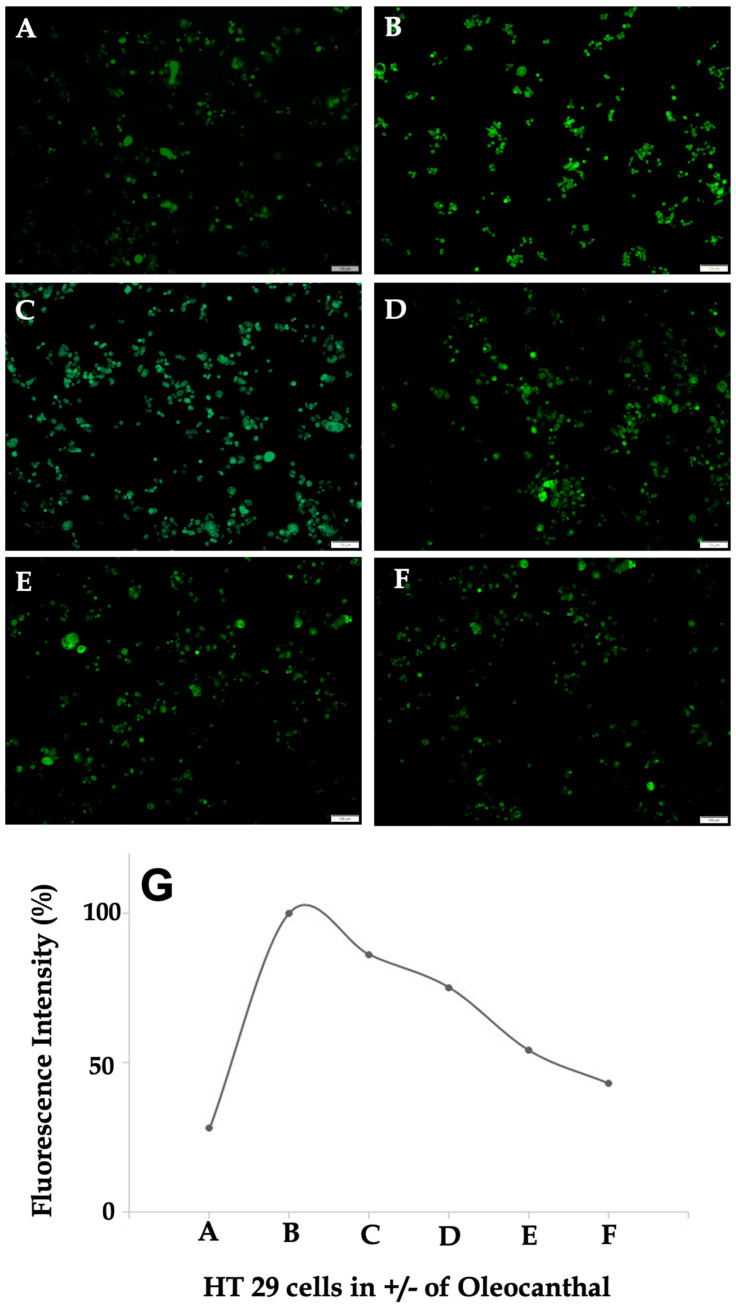
OC restores intracellular calcium levels in LPS-treated HT-29 cells. (**A**–**F**) Fluorescence images of HT-29 cells loaded with Fluo-4 to measure intracellular calcium levels under different treatment conditions. (**A**) Untreated control cells display basal fluorescence, reflecting homeostatic intracellular calcium levels. (**B**) LPS treatment (10 µg/mL) markedly increases fluorescence intensity, indicating substantial calcium mobilization via TLR4-PAR-2 activation. (**C**–**F**) Co-treatment with oleocanthal (OC) at concentrations of 20, 50, 100, and 150 µg/mL progressively reduces LPS-induced calcium flux. At 20 µg/mL (**C**), a modest reduction is evident (~20% lower than LPS alone), while at 50 µg/mL (**D**), fluorescence decreases by ~40%. Higher OC concentrations of 100 µg/mL (**E**) and 150 µg/mL (**F**) significantly restore calcium levels to near baseline, with ~65% and ~80% reductions, respectively. (**G**) Quantitative analysis of fluorescence intensity across treatment groups. Data reveal a dose-dependent attenuation of calcium mobilization by OC, with significant reductions at 50 µg/mL and greater reductions at 100 µg/mL and 150 µg/mL. Results suggest that OC effectively mitigates LPS-induced calcium dysregulation by targeting PAR-2-mediated signaling. Scale bars: 50 µm. Error bars represent standard deviation, with statistical significance denoted by compared to the LPS-treated group.

The modulatory effects of OC on intracellular calcium signaling in HT-29 cells were evaluated using the Fluo-4 assay, with results presented in Figure 5A–F and quantified in Figure 5G. Untreated control cells (Figure 5A) exhibited minimal fluorescence intensity, reflecting basal intracellular calcium levels under quiescent conditions. This homeostatic state highlights the stability of calcium signaling in the absence of inflammatory stimuli. Treatment with LPS (Figure 5B) induced a substantial increase in fluorescence intensity, demonstrating robust intracellular calcium mobilization. This effect is consistent with LPS activation of TLR4 and downstream PAR-2 signaling, which triggers phospholipase C-mediated calcium release [37]. The observed increase in fluorescence (~80% higher than the control, as shown in Figure 5G) confirms the activation of calcium-dependent pro-inflammatory pathways. Additionally, morphological alterations, including increased cellular clustering and membrane ruffling, were evident in LPS-treated cells, supporting the pro-inflammatory state induced by TLR4-PAR-2 activation. These structural changes may be indicative of heightened intracellular calcium signaling driving cytoskeletal reorganization.

Co-treatment with OC demonstrated a dose-dependent attenuation of LPS-induced calcium mobilization, as evident in Figure 5C–F. At 20 µg/mL OC (Figure 5C), a modest reduction in fluorescence intensity was observed (~20% lower than LPS alone), suggesting partial inhibition of calcium flux. This effect became more pronounced at 50 µg/mL OC (Figure 5D), where fluorescence intensity decreased by approximately 40%. At 100 µg/mL OC (Figure 5E), the calcium levels were markedly reduced, approaching baseline levels with a 65% decrease relative to the LPS-treated condition. The highest OC concentration of 150 µg/mL (Figure 5F) effectively restored calcium levels to those of the control, with fluorescence intensity reduced by ~80%. The fluorescence intensity trends presented in Figure 5G further support this progressive attenuation of calcium flux with increasing OC concentrations. The sharp peak in fluorescence at condition B (LPS alone), followed by a gradual decline with increasing OC concentrations, demonstrates the compound’s efficacy in mitigating LPS-induced calcium dysregulation. The quantitative analysis (Figure 5G) underscores this dose-dependent rescue, with statistically significant reductions observed at 50 µg/mL (*p* < 0.05) and greater reductions at 100 and 150 µg/mL (*p* < 0.01).

The results indicate that OC reduces LPS-induced calcium dysregulation in HT-29 cells and restores intracellular calcium balance in a concentration-dependent manner. This reduction in calcium flux implies a specific interruption of PAR-2-mediated calcium signaling, which plays a significant role in inflammation-related processes in CRC. Taken together, these findings suggest that OC not only counteracts LPS-mediated calcium influx but may also contribute to the stabilization of intracellular calcium homeostasis, potentially reducing downstream inflammatory transcriptional activation.

### 2.6. Effect of OC on Intracellular Calcium Levels in Caco-2 Cells

**Figure 6:**
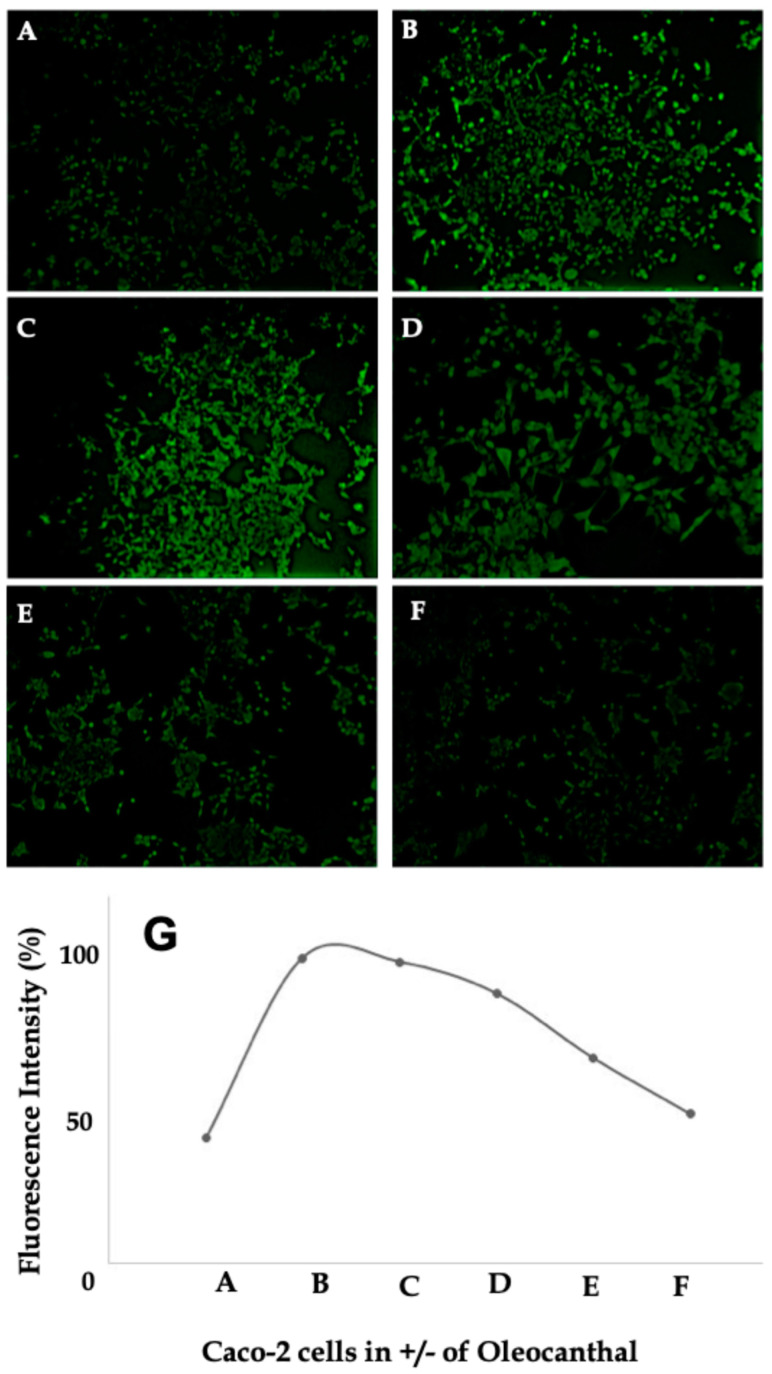
OC restores intracellular calcium levels in LPS-treated Caco-2 cells. (**A**–**F**) Fluorescence microscopy images of Caco-2 cells loaded with Fluo-4 for intracellular calcium measurement under different treatment conditions. (**A**) Control cells exhibit minimal fluorescence intensity, reflecting basal calcium levels and intact epithelial architecture. (**B**) LPS treatment (10 µg/mL) markedly increases fluorescence intensity (~80% higher than control), indicative of significant calcium mobilization via TLR4-PAR-2 signaling. LPS-treated cells display disrupted epithelial morphology, characterized by irregular borders and partial detachment. (**C**–**F**) Co-treatment with oleocanthal (OC) reduces fluorescence intensity in a dose-dependent manner and progressively restores epithelial morphology. At 20 µg/mL (**C**), modest suppression of calcium flux and partial morphological recovery are observed. At 50 µg/mL (**D**), fluorescence intensity decreases by ~40%, with notable improvement in epithelial organization. Higher OC concentrations of 100 µg/mL (**E**) and 150 µg/mL (**F**) significantly reduce calcium flux to near-control levels (~65% and ~80% reductions, respectively) and completely restore epithelial integrity. (**G**) Quantitative analysis of fluorescence intensity shows a dose-dependent reduction in calcium mobilization by OC, with significant suppression at 50 µg/mL and further reductions at 100 µg/mL and 150 µg/mL Error bars represent standard deviation. Scale bars: 50 µm. Statistical significance is denoted by compared to the LPS-treated group.

The Fluo-4 assay results presented in Figure 6A–F demonstrate the modulatory effects of OC on intracellular calcium flux in LPS-treated Caco-2 cells, providing critical insights into OC’s ability to restore calcium homeostasis under inflammatory conditions. The accompanying graph (Figure 6G) quantifies the changes in fluorescence intensity, reflecting the degree of calcium mobilization across all experimental conditions.

Caco-2 cells in the control condition (Figure 6A) exhibited minimal fluorescence intensity, indicative of basal intracellular calcium levels. The cells displayed their characteristic cobblestone morphology, with well-defined cellular borders and a cohesive epithelial arrangement, consistent with their differentiation into an enterocyte-like phenotype under noninflammatory conditions. This quiescent calcium signaling reflects the physiological integrity of untreated Caco-2 cells. The low baseline fluorescence intensity further indicates that calcium levels remain tightly regulated under homeostatic conditions, in line with their epithelial barrier function.

Upon treatment with LPS (Figure 6B), a marked increase in fluorescence intensity was observed, indicating significant calcium mobilization. This calcium flux is consistent with the activation of TLR4 by LPS, leading to PAR-2-mediated intracellular calcium release via phospholipase C. Morphologically, LPS-treated cells exhibited a disrupted epithelial architecture, characterized by irregular cellular borders, partial detachment, and evidence of stress-induced morphological changes. This loss of epithelial integrity underscores the pro-inflammatory and disruptive effects of LPS on Caco-2 cells. The fluorescence intensity increase, reaching nearly 100% relative to control (Figure 6G), underscores the extent of calcium mobilization triggered by LPS stimulation, reinforcing its role in disrupting epithelial cell function.

Co-treatment with OC demonstrated a concentration-dependent attenuation of LPS-induced calcium signaling and a progressive restoration of cellular morphology (Figure 6C–F). At 20 µg/mL OC (Figure 6C), a modest reduction in fluorescence intensity was observed compared to LPS alone. Although intracellular calcium levels remained elevated relative to the control, the epithelial organization of Caco-2 cells showed slight improvements, with partial re-establishment of cellular adherence and reduced morphological disruption. Increasing the OC concentration to 50 µg/mL (Figure 6D) further attenuated fluorescence intensity, with a notable reduction of approximately 40% compared to LPS-treated cells, as depicted in Figure 6G. The morphology of the cells showed a marked improvement, with more cohesive epithelial arrangements and decreased evidence of cellular stress. This suggests that OC’s effects extend beyond calcium regulation, potentially stabilizing epithelial junctional integrity in inflammatory conditions.

At 100 µg/mL OC (Figure 6E), fluorescence intensity was significantly reduced, nearing baseline levels observed in the control condition. This pronounced suppression of calcium flux was accompanied by a nearly complete restoration of epithelial integrity, with well-defined cellular borders and an organized epithelial monolayer. At the highest OC concentration of 150 µg/mL (Figure 6F), fluorescence intensity was nearly indistinguishable from the control condition, indicating a full rescue of LPS-induced calcium dysregulation. Additionally, the complete restoration of cellular architecture at this concentration suggests that OC mitigates calcium-associated cytotoxic stress, enabling epithelial repair.

Quantitative analysis of fluorescence intensity changes across all experimental conditions (Figure 6G) confirmed the dose-dependent effects of OC. LPS treatment alone significantly elevated fluorescence intensity by approximately 80% compared to the control (*p* < 0.05). Co-treatment with OC resulted in progressive reductions in fluorescence intensity, with statistically significant suppression observed at 50 µg/mL (*p* < 0.05) and further reductions at 100 and 150 µg/mL (*p* < 0.01). The decline in fluorescence intensity, paralleling the restoration of epithelial integrity, suggests that OC not only counteracts calcium overload but also promotes epithelial homeostasis by limiting inflammation-induced cellular stress responses. These findings underscore OC’s capacity to mitigate LPS-induced calcium mobilization and restore intracellular calcium homeostasis in Caco-2 cells while simultaneously rescuing epithelial integrity under inflammatory conditions.

### 2.7. Computational Structural Validation and Confidence Assessment of the AlphaFold-Predicted PAR-2 Model

The structural model of PAR-2 was computationally generated using AlphaFold2 [38], a deep learning-based protein structure prediction framework that integrates MSA templates, physicochemical constraints, and deep neural network-based structural inference. To evaluate the reliability and confidence of the predicted model, multiple validation metrics, including pLDDT, PAE, and IDDT scores, were analyzed systematically. These assessments enabled a quantitative and qualitative evaluation of structural accuracy, intrinsic disorder, and inter-domain flexibility within the AlphaFold-predicted conformation.

**Figure 7:**
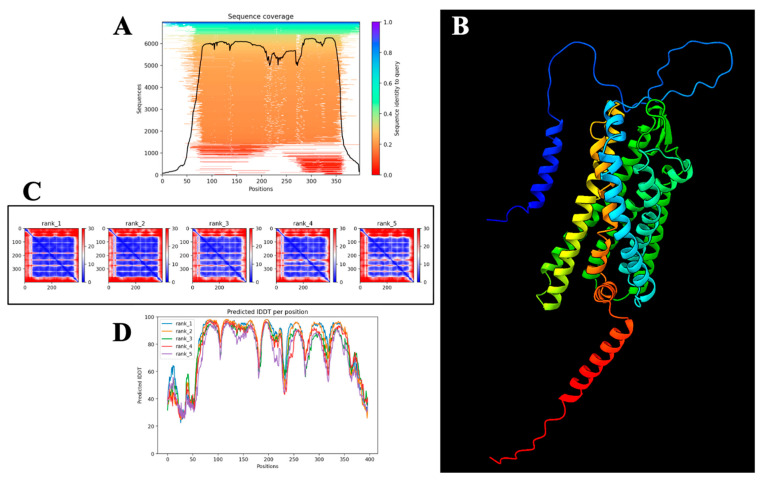
Computational modeling and confidence assessment of PAR-2 structure using AlphaFold2. (**A**) Sequence coverage plot displaying the evolutionary conservation of PAR-2 based on multiple sequence alignment (MSA). The x-axis represents amino acid positions, while the y-axis corresponds to the number of aligned sequences. The color gradient reflects sequence identity, with red indicating lower sequence conservation and blue-green representing highly conserved regions. The black trace represents the number of aligned sequences at each position, showing greater conservation within the transmembrane domains. (**B**) Structural representation of the predicted 3D model of PAR-2 visualized using ChimeraX, with the secondary structure elements distinctly highlighted. The seven transmembrane helices (TMHs) are depicted in ribbon format, color-coded from blue (N-terminal) to red (C-terminal) to emphasize the structural topology of the receptor. The extracellular and intracellular loops, as well as the disordered terminal regions, exhibit increased flexibility, consistent with lower structural confidence in these areas. (**C**) Predicted Aligned Error (PAE) matrices for the five highest-ranked AlphaFold2 models, illustrating the confidence in relative domain positioning. The blue regions correspond to low PAE values (high reliability in inter-domain positioning), while the red regions indicate increased positional uncertainty, particularly in the loop regions. (**D**) Predicted interatomic distance difference test (pIDDT) scores per residue, comparing the structural reliability of the top five AlphaFold models. Higher pIDDT values (>80) indicate structurally stable regions, predominantly within the transmembrane helices, while lower scores reflect flexibility in the extracellular and intracellular loops. (Note: This figure provides a comprehensive computational assessment of the PAR-2 model, highlighting the structural confidence of the transmembrane helices and identifying flexible loop regions that may be functionally relevant for ligand binding, receptor activation, and intracellular signaling).

The sequence coverage analysis in Figure 7A provides an overview of the extent to which homologous sequences align with the query protein across its primary sequence. The black trace overlay in the sequence coverage plot represents the number of aligned sequences contributing to each positional assignment, with regions of higher sequence identity exhibiting superior evolutionary conservation. The transmembrane domains show a high degree of sequence conservation, suggesting that these regions adopt structurally constrained and functionally indispensable motifs. Conversely, low sequence identity regions, particularly in extracellular and intracellular loops, are likely indicative of conformational plasticity, post-translational modifications, or evolutionarily acquired structural variability. These observations are consistent with prior experimental studies of GPCRs, where loop regions frequently serve as allosteric regulators of ligand binding and intracellular signaling [39].

To assess the local confidence of atomic positioning within the AlphaFold-predicted structure, pLDDT scores were analyzed (Figure 7A). The pLDDT metric, a per-residue reliability score ranging from 0 to 100, measures the model’s self-reported confidence in residue-specific spatial positioning independent of experimental constraints. Regions with pLDDT values exceeding 90 (colored in blue) are interpreted as high-confidence, well-folded domains with minimal structural ambiguity. These high-confidence regions predominantly correspond to the seven transmembrane α-helices of PAR-2, reinforcing the robustness of the helical scaffold. Intermediate confidence regions (pLDDT 70–90) correspond to extracellular and intracellular loop domains, suggesting that these regions possess partial flexibility while retaining defined secondary structures. Notably, pLDDT scores below 50 (colored in red) are observed in select terminal regions and loop segments, suggesting an increased probability of intrinsic disorder or modeling inaccuracies due to the absence of stable constraints in the training dataset.

The three-dimensional structural topology of PAR-2, as visualized in Figure 7B, using ChimeraX, provides a spatial representation of the predicted secondary and tertiary architecture. Unlike the confidence-mapped structures, this visualization primarily serves as a qualitative reference for domain organization and does not convey reliability metrics. The seven α-helices are interspersed with extracellular and intracellular loops, consistent with the canonical GPCR topology [21]. However, due to the absence of bound ligands or lipid bilayer constraints in the modeling pipeline, potential dynamic conformational states of these loops remain unaccounted for. Future studies can incorporate molecular dynamics (MD) simulations within a physiologically relevant lipid bilayer environment to capture the conformational plasticity of these loops under native-like conditions [40], thereby improving the accuracy of structural predictions.

To further evaluate the relative positioning accuracy of structural domains, PAE matrices were analyzed (Figure 7C). The PAE matrix quantifies the expected deviation (in Å) between two residue pairs within a model. Low PAE values (blue regions) indicate rigid-body-like stability between interacting regions, whereas high PAE values (red regions) suggest significant positional uncertainty. Within the PAE matrix of PAR-2, the transmembrane domains exhibit consistently low PAE scores (<5 Å), confirming their reliable spatial definition. In contrast, extracellular and intracellular loop domains show elevated PAE values (>15–25 Å), indicating increased flexibility or potential structural heterogeneity. The PAE-based inter-helical positioning is highly conserved across all five AlphaFold-generated models, further supporting the structural robustness of the helical domain.

Per-residue atomic stability was analyzed using Predicted IDDT (pIDDT) scores (Figure 7D), a metric that quantifies the confidence in residue-level interatomic distances independent of global structural constraints. Higher IDDT values (>80) correspond to structurally well-ordered residues, whereas lower values (<60) suggest localized conformational flexibility or modeling uncertainties. The pIDDT profile of PAR-2 reveals that the transmembrane helices display uniformly high stability, consistent with their expected structural rigidity within a membrane-embedded environment. Conversely, loop regions exhibit variable IDDT scores, reflecting their intrinsic flexibility, which is a hallmark of ligand-binding adaptability and intracellular signaling regulation in GPCRs.

#### Structural Reliability Assessment

The integration of pLDDT, PAE, and pIDDT scores provides a high-confidence validation of the transmembrane domain architecture, confirming that the seven-helix GPCR topology of PAR-2 is structurally well-defined and computationally robust. The low PAE values and high IDDT scores within the helical core strongly indicate that this region is a reliable structural model suitable for docking simulations and further experimental validation. However, the increased uncertainty in loop regions suggests that their conformational states may be dynamic, requiring further refinement through MD simulations or experimental structural characterization using cryo-EM or NMR spectroscopy.

Taken together, the predicted structure of PAR-2 exhibits high overall reliability, particularly within its transmembrane scaffold, making it a valid framework for structure-based drug design and ligand-binding analyses. However, loop dynamics remain an inherent limitation of the current model, necessitating additional studies to capture the functional flexibility of these regions in a physiologically relevant context.

### 2.8. Predicted Binding Mode and Structural Insights into OC Interaction with PAR-2

The docking of OC with PAR-2 was conducted to determine potential binding interactions, particularly in the context of its transmembrane localization and functional implications in CRC cells. Among the five predicted docking models, the one with the highest binding confidence and most favorable free energy score was selected for further analysis. This model revealed that OC interacts with a well-defined pocket within the transmembrane domain of PAR-2, engaging in both hydrophobic and electrostatic interactions with key residues. The identified binding pocket, which ranked as the most stable among the detected cavities, was characterized by a significant number of contact residues, including THR104, LYS106, HIS108, ALA110, VAL111, MET114, GLN172, ARG173, VAL176, ILE177, VAL178, ASN179, PRO180, MET181, GLY182, HIS183, SER184, LYS187, MET268, LEU269, SER272, ASN277, SER278, LYS281, ARG282, ARG284, ALA285, TYR345, PHE346, VAL347, SER348, HIS349, ASP350, and ARG352. These interactions suggest a stabilized ligand–receptor complex, with contributions from both hydrogen bonding and van der Waals forces.

**Figure 8:**
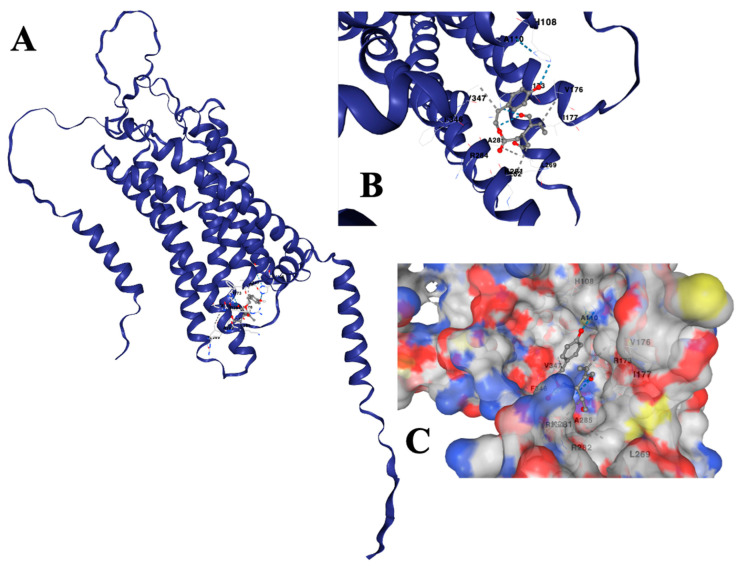
Molecular docking of oleocanthal (OC) with PAR-2 and visualization of key binding interactions. (**A**) Overall structural model of PAR-2 bound to OC, highlighting the predicted binding site within the transmembrane domain. The helical structure of PAR-2 is depicted in blue ribbon representation, with OC positioned within a well-defined ligand-binding cavity. (**B**) Close-up view of the OC-binding pocket, showing specific interactions with key amino acid residues. Hydrogen bonds and hydrophobic contacts between OC and the transmembrane residues are illustrated in dashed lines. Notable interactions include hydrogen bonding with HIS108, hydrophobic stabilization with VAL176, ILE177, and PHE346, and additional polar contacts with ARG173 and ALA285. (**C**) Molecular surface representation of the OC-binding site, emphasizing the electrostatic landscape of the binding cavity. The binding pocket is mapped according to electrostatic potential (blue: positive charge; red: negative charge), illustrating how OC interacts with key residues in the transmembrane core. The position of OC within the hydrophobic pocket suggests a stable ligand–receptor interaction, potentially influencing receptor conformation and downstream signaling. (Note: This figure provides computational evidence supporting a direct interaction between OC and PAR-2, reinforcing the hypothesis that OC may modulate PAR-2 function through direct receptor binding in addition to transcriptional downregulation. Further experimental validation through mutagenesis and binding kinetics assays is warranted to confirm these in silico findings).

The nature of these interactions is visualized in the structural representations. Figure 8A presents an overall view of the PAR-2 model with OC docked within the identified cavity. The transmembrane helices are distinctly visible, indicating the ligand’s positioning relative to the helical core. Figure 8B provides a zoomed-in depiction of the binding site, emphasizing key interacting residues and their spatial alignment with OC. Notably, polar interactions between OC and HIS108, ARG173, and TYR345 contribute to stabilizing the ligand within the cavity, while nonpolar interactions with residues such as VAL111, ILE177, and PHE346 further reinforce its placement. Figure 8C illustrates a molecular surface representation of the binding site, where OC is nestled within the transmembrane region, interacting with hydrophobic and electrostatically charged residues. The distinct surface topology highlights the accessibility of the binding site and the spatial distribution of critical residues involved in ligand recognition.

The structural model suggests that OC has a high probability of engaging directly with PAR-2 within the lipophilic environment of the membrane. However, due to the nature of PAR-2 as a transmembrane receptor, ligand accessibility is a crucial factor that must be considered. The docking results indicate that OC could potentially insert itself into the lipid bilayer before engaging with the receptor, a characteristic often observed for small-molecule modulators of GPCRs [41,42,43]. The identified binding site is located within a structurally stable region of the receptor, as inferred from the high-confidence AlphaFold model, where transmembrane residues exhibit strong pLDDT and IDDT scores, reinforcing the credibility of the predicted ligand interaction.

Despite these favorable predictions, the computational docking model requires experimental validation to confirm its biological relevance. While the observed downregulation of PAR-2 at both the protein and mRNA levels suggests a functional effect of OC, the precise mechanism—whether through direct binding or indirect regulatory pathways—remains to be elucidated. Additional structural and biochemical studies, including MD simulations, site-directed mutagenesis of the key interacting residues, and binding kinetics assays such as surface plasmon resonance or microscale thermophoresis, will be necessary to establish the physiological relevance of this interaction. While the docking model provides a theoretically feasible binding mechanism, further investigation is required to substantiate the role of OC in modulating PAR-2 signaling.

## 3. Discussion

The findings presented in this study highlight the therapeutic potential of OC as a modulator of PAR-2-mediated inflammation in CRC. This research extends the corpus of knowledge surrounding OC’s anti-inflammatory and anti-cancer properties by elucidating its selective attenuation of PAR-2 expression at both the protein and transcriptional levels in HT-29 and Caco-2 cell lines (Figure 2). The implications of these results underscore OC’s promise as a targeted therapeutic agent for inflammation-driven oncogenic pathways in CRC, particularly through its specificity in modulating PAR-2 without affecting related GPCRs, such as PAR-1 (Figure 3). This selective modulation is a crucial advantage, as broad GPCR targeting could lead to unintended systemic effects, including vascular dysfunction and coagulation disturbances.

Previous studies have demonstrated OC’s efficacy in various cancer models, including hepatocellular carcinoma (HCC), breast cancer, and CRC. OC has been shown to exert antitumor effects on HCC cells. Studies have demonstrated that OC inhibits cell viability and induces apoptosis in HCC cell lines, including HepG2, Hep3B, and PLC/PRF/5 [31]. The mechanism involves the induction of reactive oxygen species (ROS), DNA damage, and mitochondrial depolarization [31]. Notably, OC’s effects are more potent than those of traditional COX inhibitors like ibuprofen and nimesulide, and it does not affect the viability of primary normal human hepatocytes [31].

**Figure 9:**
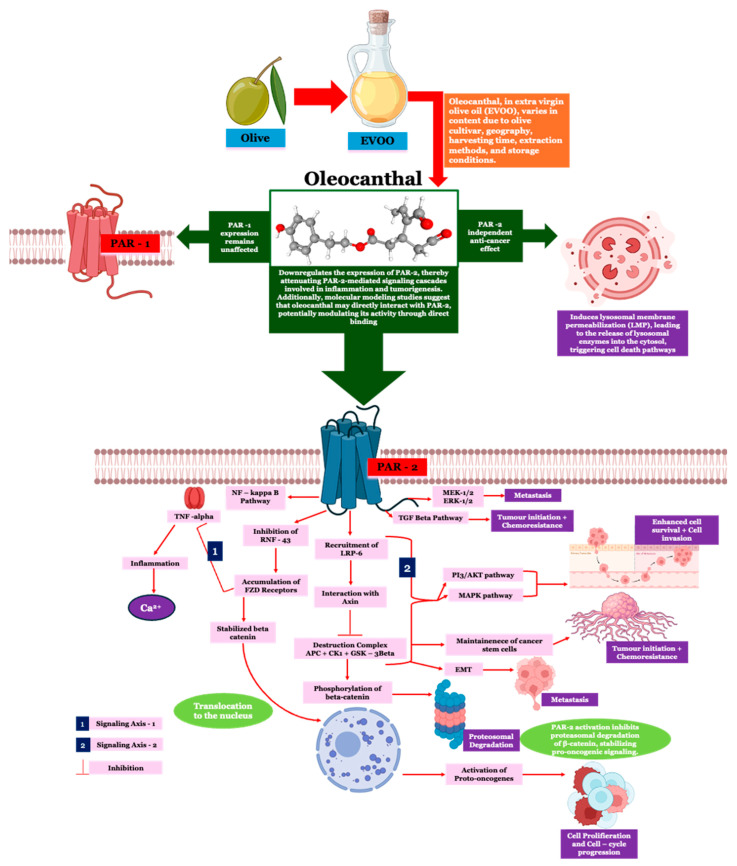
Summary of oleocanthal (OC)-mediated modulation of PAR-2 signaling in colorectal cancer (CRC). This schematic representation illustrates the multifaceted effects of oleocanthal (OC) on PAR-2-driven oncogenic pathways in colorectal cancer (CRC). **Top section**: OC, a phenolic compound derived from extra virgin olive oil (EVOO), exerts anti-inflammatory and anti-cancer effects. The concentration of OC in EVOO varies due to factors such as olive cultivar, geographic origin, harvesting time, extraction methods, and storage conditions. **Middle section**: OC selectively downregulates PAR-2 expression, while PAR-1 expression remains unaffected, reinforcing its specificity in modulating PAR-2-driven inflammation without broadly impacting GPCR signaling. Additionally, OC has PAR-2-independent anti-cancer effects, including lysosomal membrane permeabilization (LMP), which leads to lysosomal enzyme release and activation of cell death pathways. **Bottom section:** The downstream consequences of PAR-2 inhibition by OC are categorized into two key signaling axes: (1) **Signaling axis 1 (left panel)***—***inflammatory pathway suppression**: *a*. Inhibition of NF-κB signaling and TNF-α expression disrupts inflammatory cascades. *b*. Restoration of calcium homeostasis limits calcium-dependent activation of NFAT and NF-κB, further attenuating tumor-promoting inflammation. (2) **Signaling axis 2 (right panel)**—**Wnt/β-catenin and survival pathway suppression**: a. PAR-2 activation recruits LRP6, stabilizing β-catenin by preventing its proteasomal degradation. b. Inhibition of RNF-43 leads to the accumulation of FZD receptors, further enhancing Wnt signaling. c. OC-induced PAR-2 downregulation disrupts these processes, facilitating β-catenin degradation and reducing oncogenic transcription. d. Suppression of PI3K/Akt and MAPK pathways impairs tumor cell survival, epithelial–mesenchymal transition (EMT), chemoresistance, and metastasis. e. PAR-2 inhibition reduces cancer stem cell maintenance by interfering with survival signaling, decreasing tumor progression and therapy resistance. (Note: This comprehensive mechanistic model illustrates how OC’s selective inhibition of PAR-2 interferes with key oncogenic pathways involved in CRC progression, inflammation, and metastasis. Figure 9 provides an integrated overview of OC’s therapeutic impact, highlighting its potential as a novel anti-inflammatory and anti-cancer agent for CRC treatment).

In the context of breast cancer, OC has been found to suppress the growth of hormone-dependent breast cancer cells, such as BT-474, MCF-7, and T-47D [44]. It also enhances sensitivity to tamoxifen treatment. In vivo studies using BT-474 tumor xenografts in mice showed significant inhibition of tumor growth with OC treatment [44]. OC interacts with estrogen receptors, which may contribute to its anti-cancer effects in hormone-dependent breast cancers [44]. Additionally, in breast cancer cells, OC induces lysosomal membrane permeabilization (LMP), leading to the release of lysosomal enzymes into the cytosol, which triggers cell death pathways (Figure 9). This mechanism involves the translocation of galectin-3 to lysosomes, indicative of lysosomal damage [29]. However, the extent to which these findings translate to CRC remains unexplored, as estrogen receptor-mediated pathways do not play a central role in colorectal tumorigenesis.

OC has been shown to inhibit cell viability and induce apoptosis in CRC cell lines, such as HT-29 and SW480. This effect is more potent than that of traditional COX inhibitors like ibuprofen and nimesulide [31]. Furthermore, OC has been shown to exert its antitumor effects through the generation of reactive oxygen species (ROS), leading to DNA damage and mitochondrial depolarization [31]. The ROS scavenger N-acetyl-L-cysteine can suppress these effects, indicating ROS as a key mediator. The current study builds on this foundational knowledge by demonstrating OC’s dose-dependent inhibition of PAR-2, a key player in inflammation associated with CRC, thereby adding a new dimension to the therapeutic potential of OC. While ROS-mediated mechanisms of cell death have been previously reported, the present study uniquely identifies PAR-2 as a novel target of OC in CRC, expanding the mechanistic landscape of OC’s antitumor effects.

PAR-2 has a complex role in CRC, as outlined in the introduction. Its overexpression is strongly associated with aggressive tumor behavior, therapy resistance, and poor prognosis [25,45,46,47]. PAR-2 activation promotes tumor growth and metastasis through the activation of key pathways, including Wnt/β-catenin, PI3K/Akt, MAPK/ERK, and NF-κB (Figure 9). These signaling cascades are crucial mediators of epithelial–mesenchymal transition (EMT), a key step in tumor invasion and metastatic dissemination. For instance, the PAR2-LRP6-Axin axis stabilizes β-catenin, leading to the transcriptional activation of oncogenic targets such as *c-Myc* and *Cyclin D1*. Moreover, PAR-2’s inhibition of RNF43 allows for the accumulation of FZD receptors, sustaining Wnt signaling [48]. These processes are critical for maintaining cancer stem cell populations, promoting epithelial–mesenchymal transition (EMT), and fostering therapy resistance [47,49,50]. By attenuating PAR-2 expression, OC potentially disrupts these oncogenic signaling cascades, reducing the tumor-promoting microenvironment in CRC. This disruption is further evidenced by the downregulation of TNF-α expression observed in this study (Figure 4), suggesting an interruption in the NF-κB inflammatory signaling pathway [51].

Although the present study did not directly assess OC’s impact on β-catenin activity, our findings suggest several indirect mechanisms by which OC may modulate Wnt/β-catenin signaling. Given the well-documented role of Wnt signaling in CRC progression, assessing OC’s influence on this pathway remains an important future direction. The observed attenuation of PAR-2 expression, restoration of calcium homeostasis, and suppression of TNF-α collectively point to potential regulatory effects on this pathway. Moreover, considering the crosstalk between Wnt and NF-κB pathways, OC’s suppression of TNF-α could serve as an additional node of intervention in inflammation-associated tumorigenesis.

PAR-2 has been established as a stabilizer of β-catenin through interactions with LRP6 and Axin1, preventing β-catenin degradation and promoting oncogenic transcription (Figure 9) [26]. Therefore, the downregulation of PAR-2 following OC treatment likely reduces β-catenin stabilization, thereby interfering with Wnt-driven CRC progression. Furthermore, intracellular calcium signaling plays a critical role in β-catenin regulation, particularly through Ca^2+^-dependent activation of CaMKII and PKC, which enhance β-catenin nuclear translocation (Figure 9) [52]. Our demonstration that OC restores calcium homeostasis in LPS-treated CRC cells (Figure 5 and Figure 6) suggests this effect may further disrupt Wnt/β-catenin activation. Additionally, TNF-α functions as an upstream enhancer of Wnt signaling by phosphorylating Dishevelled (Dvl) and suppressing Wnt antagonists such as Dickkopf-1 (DKK1) (Figure 9) [53,54]. The reduction in TNF-α expression observed in this study (Figure 4) indicates that OC may indirectly inhibit TNF-α-driven β-catenin activation, further attenuating Wnt signaling. To validate these mechanistic links, future studies should employ β-catenin-specific reporter assays, nuclear fractionation, and Axin degradation assessments.

Calcium signaling, a downstream effect of PAR-2 activation, plays a pivotal role in inflammation-driven CRC progression [22]. Elevated intracellular calcium levels activate calcineurin, leading to the nuclear translocation of NFAT transcription factors, which drive genes associated with tumor invasion and immune evasion [55]. Furthermore, calcium signaling amplifies inflammatory pathways by enhancing NF-κB activation [56]. The results from this study demonstrate that OC mitigates LPS-induced calcium dysregulation in both HT-29 and Caco-2 cells, restoring intracellular calcium levels to baseline (Figure 5 and Figure 6). This normalization interrupts calcium-dependent inflammatory signaling, curbing the production of pro-inflammatory cytokines such as TNF-α and IL-6, thereby further reducing the pro-inflammatory milieu within the tumor microenvironment.

Thus, while OC’s impact on Wnt/β-catenin signaling was not directly evaluated, its ability to modulate upstream regulators such as PAR-2, calcium flux, and TNF-α highlights a potential mechanism through which OC may attenuate β-catenin-driven tumorigenesis. Future research should investigate the possibility of OC synergizing with established Wnt inhibitors such as LGK974 (Porcupine inhibitor), PRI-724 (β-catenin transcriptional inhibitor), or XAV939 (Tankyrase inhibitor) to enhance its therapeutic efficacy in CRC.

The specificity of OC in modulating PAR-2 without affecting PAR-1 is a notable strength of this study. PAR-1, which primarily mediates thrombin signaling, plays essential roles in normal vascular and hemostatic processes [21]. The absence of any significant modulation of PAR-1 expression by OC minimizes potential adverse effects, such as bleeding complications, that could arise from broader PAR inhibition (Figure 3). This selective modulation highlights OC’s potential as a safe and effective therapeutic agent, particularly in CRC patients with inflammation-driven tumorigenesis.

Dietary studies have further highlighted the benefits of OC as a component of the Mediterranean diet. For instance, long-term consumption of extra virgin olive oil, rich in OC, has been associated with reduced CRC incidence and improved overall survival in patients with inflammatory bowel disease [57,58,59]. These effects are attributed to OC’s ability to modulate gut microbiota [60], enhance epithelial barrier integrity [61], and reduce systemic inflammation [62], thereby creating a less favorable environment for tumorigenesis. Variability in OC content in commercial olive oil has been documented, with studies showing a range between 200 and 700 mg/L depending on factors such as olive variety, geographic origin, and extraction methods [63]. This highlights the importance of standardizing OC levels in dietary interventions to ensure therapeutic efficacy.

In this study, we observed that OC significantly downregulates PAR-2 expression at both the transcriptional (mRNA) and protein levels in CRC cells. However, PAR-1 expression remained relatively unchanged, suggesting that OC selectively targets PAR-2 without broadly suppressing GPCR signaling. While this finding establishes OC as a promising modulator of PAR-2-driven inflammation, the precise molecular mechanisms underlying its downregulatory effect remain to be fully elucidated. Based on the regulatory pathways governing G-protein-coupled receptors (GPCRs) and inflammation-driven oncogenic signaling, several plausible mechanisms can explain this effect. *Firstly*, OC may suppress PAR-2 expression at the transcriptional level by interfering with key transcription factors and epigenetic regulators involved in PAR-2 gene regulation. PAR-2 transcription is known to be modulated by inflammatory transcription factors such as NF-κB [24], AP-1 [64], and STAT3 [65], all of which are strongly implicated in CRC progression and inflammation [66,67]. Previous studies have shown that OC attenuates NF-κB nuclear translocation and inhibits its DNA-binding activity, thereby reducing the transcription of pro-inflammatory genes [68]. Since PAR-2 is an NF-κB-responsive gene, the suppression of NF-κB signaling by OC may indirectly lead to decreased PAR-2 transcription. Additionally, OC has been reported to modulate histone acetylation and DNA methylation patterns [69], which could further alter the chromatin accessibility of the *F2RL1* gene (encoding PAR-2), contributing to its transcriptional repression. Importantly, this transcriptional downregulation appears to be selective for PAR-2, as PAR-1 expression was unaffected, indicating that OC does not broadly interfere with GPCR transcription but rather exerts gene-specific effects.

*Secondly*, OC may promote the degradation of PAR-2 protein through proteasomal or lysosomal pathways. GPCRs such as PAR-2 are tightly regulated at the post-translational level, where receptor internalization, ubiquitination, and degradation govern their stability and turnover. One well-characterized mechanism of PAR-2 downregulation involves ubiquitin-mediated proteasomal degradation, which is often triggered by desensitization following receptor activation [70]. Alternatively, lysosomal degradation pathways, particularly those regulated by G-protein-coupled receptor kinases (GRKs) and β-arrestins, have been shown to regulate PAR-2 levels under inflammatory conditions [71]. Since PAR-1 levels were not affected by OC, this suggests that OC does not cause generalized GPCR degradation but may selectively accelerate PAR-2-specific degradation pathways, potentially through enhanced ubiquitination or β-arrestin-mediated internalization. Future studies employing ubiquitin immunoprecipitation assays, proteasome inhibition (e.g., MG132), and lysosomal inhibitors (e.g., chloroquine or bafilomycin A1) could help delineate the precise contribution of these pathways to OC-mediated PAR-2 downregulation.

*Thirdly*, OC may interfere with the stability of PAR-2 mRNA through post-transcriptional regulatory mechanisms. The stability of PAR-2 mRNA is controlled by RNA-binding proteins (RBPs) and microRNAs (miRNAs) that modulate transcript half-life and degradation rates [72,73]. Given that inflammatory conditions stabilize the mRNAs of various pro-inflammatory mediators [74], it is plausible that PAR-2 mRNA stability may also be enhanced, thereby sustaining inflammatory signaling. OC may counteract this effect by either suppressing stabilizing RBPs (such as HuR) or enhancing the activity of miRNAs that target the PAR-2 transcript. This would lead to accelerated mRNA degradation, contributing to the observed reduction in PAR-2 expression at the mRNA level. Investigating changes in PAR-2 mRNA half-life following OC treatment using actinomycin D chase assays could help determine whether this mechanism is involved.

*Lastly*, OC may directly interact with PAR-2 and modulate its activity at the receptor level. Given OC’s lipophilic structure and phenolic backbone, there is a possibility that OC may bind directly to PAR-2, thereby influencing its function or stability. To further investigate this possibility, we conducted molecular docking studies using the AlphaFold-predicted structure of PAR-2 as a receptor model (Figure 7). The docking simulations, performed using CB-Dock2, identified a high-confidence binding pocket within the transmembrane domain of PAR-2, suggesting a direct interaction between OC and the receptor (Figure 8).

Among the five docking poses generated, the top-ranked model exhibited a favorable binding energy, indicating a stable ligand–receptor complex. Key interactions were observed between OC and several critical amino acid residues within the transmembrane domain, including HIS108, ARG173, TYR345, and PHE346, which contribute to ligand stabilization via hydrogen bonding and hydrophobic interactions (Figure 8A,B). Additionally, VAL111, ILE177, and MET181 were found to participate in nonpolar interactions, further reinforcing OC’s binding affinity (Figure 8A,B).

These computational findings suggest that OC may exert its effects by binding within a structurally conserved region of PAR-2, potentially influencing receptor stability or trafficking. However, while these docking results provide theoretical support for OC’s ability to engage with PAR-2, experimental validation is essential to confirm its biological relevance. Techniques such as MD simulations, site-directed mutagenesis of key interacting residues, and biophysical binding assays (e.g., surface plasmon resonance and microscale thermophoresis) could help substantiate these computational predictions.

By integrating in silico modeling with our in vitro findings, we propose that OC may modulate PAR-2 activity through a combination of direct receptor interaction and transcriptional downregulation. Future investigations should focus on validating these interactions experimentally and delineating their functional consequences in PAR-2-mediated inflammatory signaling in CRC.

## 4. Materials and Methods

### 4.1. Study Design

This investigation was conceptualized as a cross-sectional in vitro experimental study, meticulously designed to delineate the immediate effects of OC on the modulation of inflammatory mediators within CRC cell lines. The cross-sectional framework enabled the precise interrogation of OC’s influence on key inflammatory signaling pathways at a singular temporal juncture, thereby offering a comprehensive yet temporally constrained elucidation of its mechanistic impact. By obviating the necessity for longitudinal or iterative analyses, this methodological approach provided a cogent and incisive assessment of OC’s therapeutic potential as a functional nutraceutical, underscoring its capacity to attenuate inflammation within the pathophysiological context of CRC.

### 4.2. Ethics Considerations

The experimental procedures in this study were exclusively conducted in vitro, utilizing commercially available cell lines, and did not involve the use of animal models, patient-derived samples, or human subjects. Accordingly, the research is categorized as minimal risk and qualifies for exempt review under the regulations of the Institutional Review Board (IRB) at Mohammed Bin Rashid University of Medicine and Health Sciences (MBRU). For further information or clarification, correspondence may be directed to the MBRU IRB at irb@mbru.ac.ae.

No individuals, including minors, were involved in this study, thereby obviating the need for parental or guardian consent. Furthermore, the research did not necessitate access to medical records, archived samples, or any form of direct or indirect interaction with human participants.

In summary, this investigation was strictly limited to in vitro experimentation using established cell lines, ensuring full compliance with ethical standards. Consequently, the requirement for informed consent was waived.

### 4.3. Cell Line Selection

To model distinct stages of CRC, we employed the HT-29 and Caco-2 cell lines, each selected for their unique biological characteristics. HT-29 cells, derived from a grade II adenocarcinoma, exemplify advanced CRC due to their aggressive proliferative capacity and pronounced epithelial–mesenchymal transition (EMT) potential, rendering them particularly suited for the investigation of metastasis and PAR-2-mediated signaling pathways [75,76,77]. Conversely, the Caco-2 cell line, originating from a well-differentiated adenocarcinoma, serves as a model for early-stage CRC [78]. Its capacity to undergo differentiation into enterocyte-like cells provides an excellent platform for elucidating the role of PAR-2 in tumor initiation and progression [79,80]. Together, these cell lines constitute a complementary system, facilitating the comprehensive evaluation of therapeutic interventions targeting PAR-2-mediated inflammation across the continuum of CRC stages.

Given the focus of this study on PAR-2-driven inflammatory pathways, it was imperative to employ cell lines with demonstrably high PAR-2 expression. As no comprehensive dataset comparing PAR-2 expression across multiple CRC cell lines was available, we initially referred to the study by Darmoul et al., which presented Northern blot data on PAR-2 mRNA levels across various CRC models [34]. To ensure a quantitative assessment, we performed a densitometric analysis of the Northern blot signal intensities, normalizing PAR-2 expression to GAPDH levels. These results, now presented as Figure 1A in this study, confirm that HT-29 and Caco-2 cells exhibit the highest PAR-2 expression among the evaluated CRC cell lines, thereby reinforcing their selection.

While T84 cells also displayed relatively high PAR-2 expression, they were excluded due to their slow proliferation rate, confluency-dependent differentiation, and preferential use in epithelial barrier and ion transport studies rather than in inflammatory or oncogenic models [81,82]. Similarly, SW480 was not selected despite moderate PAR-2 expression, as it originates from a primary tumor rather than a metastatic site [83], making it less representative of invasive and metastatic CRC phenotypes, where PAR-2 activity is expected to be more pronounced [84]. Additionally, SW480 cells exhibit lower inflammatory cytokine responsiveness compared to HT-29, making them a suboptimal model for studying PAR-2-driven inflammatory pathways [85].

HCT-8 cells, despite their established role in drug resistance and CRC migration models, were not chosen due to their inherently low PAR-2 expression, as confirmed by Figure 1A. Additionally, HCT-8 cells lack the well-characterized differentiation profiles of HT-29 and Caco-2, limiting their utility in investigating early- to late-stage CRC progression [86,87].

Cl.19A cells, although used in select CRC studies, were not included due to their highly variable differentiation potential and lower reproducibility in inflammatory assays [88,89]. These cells, derived from a more heterogeneous CRC subpopulation, exhibit considerable variability in gene expression and phenotypic plasticity. This variability introduces inherent inconsistencies in inflammatory pathway activation, including PAR-2 signaling.

Unlike well-characterized epithelial CRC models such as HT-29 and Caco-2, which maintain relatively stable molecular and phenotypic profiles, Cl.19A cells display pronounced fluctuations in epithelial and mesenchymal markers. These fluctuations are influenced by culture conditions, passage numbers, and environmental stimuli. Such variability complicates the interpretation of inflammatory responses, particularly in PAR-2-driven cascades that rely on tight regulatory control of downstream transcriptional programs.

Studies have demonstrated that intratumoral heterogeneity in CRC is often driven by transcriptional plasticity rather than stable genetic alterations, leading to dynamic changes in gene expression profiles within tumor cell populations. This plasticity can result in variable responses to inflammatory stimuli and therapeutic agents, thereby posing challenges for controlled investigations of specific signaling pathways [90].

In contrast, HT-29 and Caco-2 cell lines have been extensively characterized and are known for their stable expression of epithelial markers and consistent phenotypic traits. HT-29 cells, for instance, can differentiate into mature intestinal cells expressing brush border-associated hydrolases, forming monolayers with tight junctions and a typical apical brush border [91]. Similarly, Caco-2 cells spontaneously differentiate into enterocyte-like cells, making them reliable models for studying intestinal epithelial function and associated signaling pathways [78].

Thus, the selection of HT-29 and Caco-2 cells was guided by both their biological relevance to CRC progression and their robust PAR-2 expression profile, ensuring that the findings of this study remain mechanistically sound and translationally applicable.

### 4.4. Cell Culture and Treatment

For all experiments, HT-29 and Caco-2 cells were seeded at a density of 1 × 10^7^ cells per well to ensure uniform experimental conditions. This cell density was chosen based on established in vitro protocols investigating CRC inflammation and signaling pathways [92,93]. All experiments were conducted in biological triplicates (n = 3), with each independent experiment including technical replicates, and statistical analyses were performed to validate reproducibility and minimize variability.

### 4.5. Cell Culture Methodology

The HT-29 and Caco-2 cell lines were cultured under rigorously sterile conditions to uphold experimental fidelity. Cryopreserved cells were rapidly thawed in a 37 °C water bath and gently transferred into sterile 15 mL conical tubes containing 10 mL of pre-warmed complete culture medium. HT-29 cells were maintained in Roswell Park Memorial Institute (RPMI) 1640 medium supplemented with 10% fetal bovine serum (FBS), 1% penicillin–streptomycin, and 1% L-glutamine. In contrast, Caco-2 cells were cultured in Dulbecco’s modified eagle medium (DMEM) High Glucose medium, enriched with 10% FBS, 1% penicillin–streptomycin, and 1% nonessential amino acids. Following centrifugation at 200× *g* for 5 min, the supernatant was aspirated, and cell viability was assessed via Trypan Blue exclusion. Cells were seeded at a density of 1 × 10^7^ cells/mL in T-75 culture flasks and incubated at 37 °C in a humidified atmosphere of 5% CO_2_. Growth was monitored daily via microscopic observation to ensure morphological integrity and the absence of contamination. The culture medium was replenished every 2–3 days, with cells sub-cultured upon reaching 70–80% confluence, typically within 2–3 days, to maintain optimal growth conditions.

### 4.6. Subculturing Protocol

Upon reaching 70–80% confluence, cells were prepared for subculturing to maintain optimal growth conditions. The growth medium was aspirated, and cells were gently washed with sterile phosphate-buffered saline (PBS) to remove residual medium and metabolic byproducts. Subsequently, 2 mL of 0.25% Trypsin-EDTA (Sigma, St. Louis, MO, USA) was added to enzymatically detach the cells, with incubation at 37 °C for 2–4 min. Detachment was carefully monitored under a microscope to prevent over-trypsinization. Trypsin activity was neutralized with an equal volume of complete growth medium. The cell suspension was centrifuged at 200× *g* for 5 min, and the resulting pellet was resuspended in fresh medium. Cells were then seeded into new T-75 culture flasks at a 1:3 ratio and incubated at 37 °C in a humidified atmosphere containing 5% CO_2_ to ensure continued proliferation.

### 4.7. Cryopreservation of HT-29 and Caco-2 Cells

Cryopreservation was employed to secure long-term storage of HT-29 and Caco-2 cells while preserving their viability and functionality upon thawing. Once cells reached 70–80% confluence, they were enzymatically detached using 0.25% Trypsin-EDTA, as described above. Detached cells were centrifuged at 200× *g* for 5 min to form a pellet, which was subsequently resuspended in a cryoprotective medium containing 5% dimethyl sulfoxide (DMSO) to mitigate ice crystal formation during freezing. The cell density was adjusted to 1 × 10^6^ cells/mL, and aliquots were transferred into sterile cryovials. A controlled-rate freezing container was used to achieve gradual cooling at a rate of −1 °C per minute, ensuring minimal thermal shock. Cryovials were then transferred to liquid nitrogen storage for long-term preservation, maintaining cellular integrity and reproducibility for future experimental use.

### 4.8. Assessment of Cytotoxicity Using the MTT Assay

The cytotoxic potential of LPS was assessed prior to its application for inducing inflammation in HT-29 and Caco-2 cells, employing the 3-(4,5-dimethylthiazol-2-yl)-2,5-diphenyltetrazolium bromide (MTT) assay (Thermo Fisher Scientific, Waltham, MA, USA) in accordance with established protocols [94]. Following treatment of HT-29 and Caco-2 cells with varying concentrations of LPS, cells were incubated with MTT solution to allow enzymatic reduction in MTT to formazan crystals by metabolically active cells. The resulting formazan was solubilized, and optical density was quantified spectrophotometrically at a wavelength of 570 nm. Cell viability was calculated as a percentage relative to untreated controls, providing a reliable measure of LPS-induced cytotoxicity and ensuring appropriate dosing for subsequent inflammatory induction experiments.

### 4.9. Inducing Inflammation in HT-29 and Caco-2 Cells Using LPS and OC Treatment

To emulate a pro-inflammatory environment in CRC models, LPS was administered at a concentration of 10 µg/mL to HCT-29 and Caco-2 cells. This concentration was selected to activate Toll-like receptor 4 (TLR4) signaling, initiating downstream cascades such as the NF-κB pathway and leading to the secretion of pro-inflammatory cytokines, particularly TNF-α [95]. The use of this dose aligns with well-established protocols [94] and avoids overstimulation and excessive inflammation, which could compromise experimental fidelity [95].

Beyond its direct effects on TLR4 signaling, LPS was deliberately chosen as the inflammatory stimulus for this study due to its ability to drive endogenous PAR-2 activation in a manner that closely mirrors inflammatory conditions observed in CRC. While PAR-2 is canonically activated by serine proteases such as trypsin or mast cell tryptase, increasing evidence suggests that PAR-2 activation in inflammatory disease states is often secondary to TLR4-driven protease upregulation rather than direct proteolysis [96,97]. LPS exposure has been demonstrated to upregulate endogenous PAR-2 activators, including tryptase-β [98], neutrophil elastase [99], and matrix metalloproteinase-9 (MMP-9) [100], thereby facilitating indirect PAR-2 activation through protease release in epithelial and immune cells. Additionally, LPS-induced NF-κB activation primes PAR-2 expression, sensitizing cells to subsequent protease-mediated activation and amplifying inflammatory signaling. Given the well-established bidirectional TLR4-PAR-2 signaling axis in colorectal carcinogenesis and colonic inflammation, LPS administration represents a physiologically relevant experimental paradigm for investigating PAR-2-mediated inflammatory signaling networks within the tumor microenvironment [96,101].

Alternative methodologies for direct PAR-2 activation, including pancreatic trypsin or synthetic PAR-2-activating peptides (PAR2-APs) such as SLIGRL-NH_2_, were determined to be suboptimal for this experimental system due to multiple mechanistic and methodological constraints. Trypsin, while recognized as an endogenous PAR-2 agonist, exhibits promiscuous proteolytic activity toward multiple protease-activated receptors, including PAR-1, PAR-3, and PAR-4 at supraphysiological concentrations, thereby confounding receptor-specific signal transduction analysis [102,103,104]. Moreover, trypsin-induced PAR-2 activation proceeds via irreversible proteolytic cleavage of the N-terminal extracellular domain, resulting in rapid receptor desensitization, β-arrestin-mediated internalization, and subsequent lysosomal degradation—phenomena that inadequately recapitulate the sustained inflammatory activation characteristic of colorectal carcinoma [105,106,107,108]. Furthermore, trypsin bioactivity is rapidly neutralized by endogenous serine protease inhibitors, including α1-antitrypsin and α2-macroglobulin, significantly limiting its stability and pathophysiological relevance in models of chronic inflammation [109].

Similarly, the synthetic hexapeptide SLIGRL-NH_2_, despite selectively activating PAR-2 through nonproteolytic mechanisms, lacks pathophysiological relevance within the context of colorectal carcinoma-associated inflammation. Unlike bacterial lipopolysaccharide, which mimics microbiota-derived endotoxin exposure, SLIGRL-NH_2_ fails to engage endogenous inflammatory cascades and has demonstrated considerable off-target activity at Mas-related G-protein-coupled receptors (Mrgprs) expressed on immunocompetent cells, further complicating its application in inflammation-focused investigations [110]. Additionally, SLIGRL-NH_2_ demonstrates poor metabolic stability in biological matrices due to rapid aminopeptidase-mediated hydrolysis, necessitating frequent supplementation and introducing experimental variability in receptor activation kinetics [111]. Most significantly, SLIGRL-NH_2_ fails to induce critical inflammatory transcriptional programs, including canonical NF-κB activation and subsequent TNF-α biosynthesis, both of which are integral to our investigation of inflammation-mediated PAR-2 signaling pathways [112].

Given these critical considerations, LPS administration as the inflammatory stimulus confers distinct experimental advantages in recapitulating the endogenous mechanisms through which PAR-2 is activated during states of chronic inflammation. LPS exposure not only sustains protease-mediated PAR-2 activation but also faithfully reproduces the long-term inflammatory dynamics observed in colorectal carcinoma, wherein microbiota-derived endotoxins perpetuate chronic inflammatory states via TLR4-PAR-2 signaling crosstalk [101]. This experimental approach is further substantiated by studies demonstrating that LPS stimulation enhances PAR-2 transcriptional expression, culminating in prolonged receptor activation and pro-inflammatory cytokine production [96]. Moreover, PAR-2 genetic ablation models have exhibited attenuated inflammatory responses following the LPS challenge, providing compelling evidence for the synergistic interaction between TLR4 and PAR-2 in inflammatory signal transduction [113].

Therefore, LPS was selected as the primary inflammatory stimulus in this investigation to ensure that PAR-2 activation occurs within a physiologically relevant inflammatory microenvironment rather than through artificial and transient activation modalities using trypsin or synthetic PAR2-APs. The implementation of LPS as an inflammatory modulator enhances the translational significance of our findings, ensuring that our experimental system accurately models colorectal carcinoma-associated inflammation with appropriate mechanistic fidelity.

OC was introduced 24 h post-LPS exposure at concentrations of 20, 50, 100, and 150 µg/mL. The dose range was derived from the structural and functional parallels of OC to NSAIDs, particularly ibuprofen, which exhibits dose-dependent modulation of inflammatory mediators in cellular models [114]. The selection of 20 and 50 µg/mL was guided by the hypothesis that these concentrations represent the threshold required for COX inhibition, thereby mitigating pro-inflammatory signaling cascades. This hypothesis is supported by OC’s established biochemical interactions with molecular pathways known to be targeted by NSAIDs, particularly through its impact on COX activity and NF-κB modulation [28]. The inclusion of 100 and 150 µg/mL as higher concentrations was necessitated by evidence demonstrating that OC exerts potent anti-inflammatory and pro-apoptotic effects at these levels in cancer models. Previous studies have shown that OC at these concentrations effectively attenuates inflammatory cytokine secretion, disrupts NF-κB-mediated transcriptional activation, and induces apoptosis via intrinsic and extrinsic caspase activation pathways [30]. These concentrations are also within an order of magnitude of clinically relevant OC exposure, particularly when considering localized gastrointestinal concentrations prior to systemic metabolism.

A recent clinical trial (NCT04215367) investigating the effects of OC-rich EVOO in patients with Chronic Lymphocytic Leukemia (CLL) provides empirical evidence regarding the physiological intake of OC [115]. In this study, participants consumed 40 mL of high-OC EVOO daily, which contained 416 ± 7 mg/kg of OC. Given the approximate density of EVOO (~920 mg/mL), this corresponds to an OC concentration of ~452 µg/mL, resulting in a daily OC intake of ~18.08 mg per individual.

Moreover, the selection of OC concentrations (20–150 µg/mL) in this study is further validated by prior research investigating its biological activity across multiple therapeutic targets. Notably, OC exhibits potent inhibition of COX-2 with an IC50 of 0.21 µg/mL, surpassing the inhibitory efficacy of ibuprofen at equivalent concentrations [116]. This inhibition is central to its anti-inflammatory activity, as COX-2 is a key enzyme in pro-inflammatory prostaglandin biosynthesis. In oncogenic pathways, OC inhibits c-Met phosphorylation, a critical regulator of tumorigenesis, with an IC50 of 1.5–1.6 µg/mL, leading to suppression of the PI3K/Akt and MAPK signaling cascades [28,117]. Furthermore, OC demonstrates selective cytotoxicity in cancer cell lines, with IC50 values ranging from 3.4 to 7.9 µg/mL depending on the cancer subtype, while sparing normal epithelial cells [117]. Given these findings, the lower OC concentrations (20–50 µg/mL) used in this study align with the threshold for COX inhibition and modulation of inflammatory signaling. The higher OC concentrations (100–150 µg/mL) correspond with effective suppression of oncogenic pathways and apoptosis induction, as demonstrated in prior studies on breast and colorectal cancer models.

Further reinforcing the physiological relevance of the selected in vitro concentrations, the concentration of OC in commercial EVOO varies widely, ranging from 0.18 µg/mL to 458 µg/mL, depending on multiple factors affecting its composition [116,118,119,120]. These include the following:

(a) Olive cultivar: certain cultivars, such as Coratina, exhibit substantially higher OC concentrations (78.2 µg/mL), whereas others, such as Taggiasca, contain significantly lower levels (8.3 µg/mL);
(b) Geographical location: Italian EVOOs have been reported to contain up to 191.8 µg/mL of OC, whereas EVOOs from the United States typically exhibit lower levels (~22.6 µg/mL);
(c) Agricultural techniques: increased irrigation reduces OC content, suggesting that water stress conditions enhance OC biosynthesis;
(d) Olive maturity and harvest time: OC concentration is dependent on the degree of ripeness at harvest, with early harvests yielding EVOO with higher phenolic content;
(e) Processing methods: EVOO extraction techniques influence OC retention, with two-phase centrifugation preserving higher OC levels compared to three-phase methods;
(f) Storage conditions and thermal stability: the chemical stability of OC is affected by exposure to oxygen, light, and temperature fluctuations, though OC remains relatively stable when initially present in high concentrations in EVOO.

These insights substantiate that OC intake through dietary sources is within a physiologically plausible range for exerting anti-inflammatory and tumor-suppressive effects in colorectal cancer models, particularly within the gastrointestinal lumen, where local OC concentrations may exceed systemic plasma levels before undergoing metabolic conversion. By employing this dose range, this study comprehensively evaluates both the minimum effective concentration required to elicit an anti-inflammatory response and the upper threshold at which OC exerts maximal therapeutic efficacy in modulating PAR-2-driven inflammatory signaling.

### 4.10. Assessment of TNF-α Secretion Using ELISA

Supernatants from treated and untreated HT-29 and Caco-2 cell cultures were harvested to evaluate TNF-α secretion. Following incubation, the supernatants were centrifuged at 3000 rpm for 10 min at 4 °C to ensure the removal of cellular debris and obtain clarified samples. TNF-α levels were quantitatively measured using a commercially available enzyme-linked immunosorbent assay (ELISA) kit (Abcam, Waltham, MA, USA), meticulously adhering to the manufacturer’s protocol. Microtiter plates pre-coated with anti-TNF-α capture antibodies facilitated the selective detection of the cytokine. Optical densities were determined at 450 nm, with 620 nm as the reference wavelength, using a Hidex microplate reader. This approach enabled precise quantification of TNF-α secretion in the cell supernatants, providing critical insights into the inflammatory response before and after experimental treatments.

### 4.11. Western Blot Analysis for the Expression of PAR-1 and PAR-2

Western blot analysis was employed to evaluate the expression of PAR-1 and PAR-2 in HT-29 and Caco-2 cells following treatment with OC. PAR-1 was included as a control to determine the specificity of OC’s effects on PAR-2. Both PAR-1 and PAR-2, members of the protease-activated receptor (PAR) family, share significant structural homology, including GPCR architecture and activation by proteolytic cleavage [21]. However, their functional roles diverge: PAR-1 is primarily activated by thrombin [51,121], whereas PAR-2 is activated by trypsin-like proteases (referenced in the introduction). Moreover, to objectively determine PAR-1 as the relevant comparator, we conducted pairwise sequence alignments using Clustal Omega (Version 1.2.4) and Jalview (Version 2.11.4.1), revealing the following sequence identities with PAR-2: PAR-2 vs. PAR-1 → 34.25% identity; PAR-2 vs. PAR-3 → 34.9% identity; PAR-2 vs. PAR-4 → 32.15% identity. Despite PAR-3 exhibiting slightly higher sequence similarity, it primarily functions as a regulatory receptor that modulates the activity of PAR-1 and PAR-4 rather than being an independent driver of tumorigenesis [122]. While PAR-4 contributes to CRC progression by promoting cell proliferation, survival, and metastasis, its role is considered secondary to that of PAR-2 and PAR-1. This is due to the more prominent involvement of PAR-2 and PAR-1 in CRC progression, as evidenced by their overexpression in tumor-associated fibroblasts and their significant roles in tumor growth and invasion [35,123]. In contrast, PAR-4’s expression is often absent in normal colon mucosa but becomes evident in dysplastic and cancerous tissues, suggesting a role in tumor progression. However, the extent of PAR-4’s influence on CRC progression is less pronounced compared to PAR-2 and PAR-1, which are more directly implicated in promoting tumor growth, invasion, and metastasis [124]. Given this functional significance, PAR-1 was selected as the most appropriate comparator to determine whether OC’s effects were specific to PAR-2 or extended to other oncogenic GPCRs.

#### 4.11.1. Protein Extraction and Quantification

Cells were lysed in an ice-cold lysis buffer (0.5% SDS, 50 mM Tris-HCl, pH 7.4, supplemented with protease and phosphatase inhibitor cocktails (Roche, Basel, Switzerland)) to prevent post-lysis degradation and dephosphorylation. Lysates were centrifuged at 12,000× *g* for 15 min at 4 °C, and the supernatants were collected for protein quantification using the Bicinchoninic Acid (BCA) assay (Thermo Fisher Scientific, Waltham, MA, USA). Protein concentration was measured in duplicate using a Hidex microplate reader (Turku, Finland) at 562 nm absorbance, and sample concentrations were standardized for downstream analysis.

#### 4.11.2. Experimental Design

HT-29 and Caco-2 cells were treated with OC at concentrations of 20, 50, 100, and 150 µg/mL after LPS-induced inflammation. GAPDH was used as a housekeeping protein due to its stable expression in CRC cells across experimental conditions [125,126,127]. Protein samples (20 µg per lane) from treated and untreated cells were resolved on 10% SDS-PAGE, and a pre-stained protein ladder (Thermo Fisher, 26616) was used for molecular weight verification. Transferred onto 0.45-µm nitrocellulose membranes (Bio-Rad Laboratories, Mississauga, ON, Canada) at 4 °C for subsequent analysis.

#### 4.11.3. Blocking and Antibody Incubation

Membranes were blocked for 1 h at 4 °C with gentle rocking with 3% bovine serum albumin (BSA) in Tris-Buffered Saline (TBS, Pierce, Waltham, MA, USA) to prevent nonspecific binding. Following blocking, membranes were washed with TBS containing 0.1% Tween-20 (TTBS). Primary antibodies against PAR-1 and PAR-2 (Santa Cruz Biotechnology, Dallas, TX, USA) were diluted 1:1000 in SuperBlock (Thermo Fisher, diluted 1:10 in TTBS) and incubated overnight at 4 °C.

#### 4.11.4. Secondary Antibody and Detection

After three washes with TTBS, membranes were incubated with a 1:2000 dilution of anti-mouse IgG-HRP secondary antibody for 1 h at room temperature. Protein bands were visualized using the SuperSignal ULTRA Chemiluminescent Substrate (Pierce) and captured on Kodak Biomax photographic film (GE Healthcare, Mississauga, ON, Canada). To minimize nonspecific binding, membranes were washed three times for 10 min each in TBS before secondary antibody incubation.

#### 4.11.5. Quantification

The intensity of PAR-2 protein bands was quantified using ImageJ software (Version 1.54) (NIH, Bethesda, MD, USA), with densitometric analysis performed using the integrated density function [128]. To ensure accuracy, background correction was applied to each band using the following equation:

Corrected IntDen = RawIntDen_band − (Area_band × Mean background),

where RawIntDen_band represents the sum of pixel intensities within the band’s Region of Interest (ROI), Area_band corresponds to the size of the ROI in pixels, and Mean background denotes the average intensity of three adjacent background regions.

Normalization was carried out using GAPDH as a loading control, and all values were expressed as relative fold-change against untreated controls, which were set at 1.0. The linearity of GAPDH expression across protein loads (5–50 µg) was confirmed, ensuring its suitability as a reference control. To validate the reproducibility of the data, the coefficient of variation (CV) of GAPDH intensities across replicates was maintained below 15%, confirming consistency in protein loading. Statistical analysis was conducted using GraphPad Prism 9.0 (GraphPad Software, La Jolla, CA, USA), with data presented as mean ± standard error of the mean (SEM) for triplicate experiments (n ≥ 3). Multi-group comparisons were performed using one-way ANOVA followed by Tukey’s post hoc test [129], with a significance threshold set at *p* < 0.05.

### 4.12. RNA Extraction and cDNA Synthesis

Total RNA was extracted from both control and inflammatory (LPS-treated) HT-29 and Caco-2 cells before and after treatment with OC at concentrations of 20, 50, 100, and 150 µg/mL, using the Total RNA Isolation Kit (NZYTech, Lisbon, Portugal). The quality and integrity of the RNA were meticulously assessed using a Nanodrop Spectrophotometer (Thermo Scientific, Waltham, MA, USA), ensuring consistent purity and concentration across all experimental samples. Only RNA samples exhibiting A260/A280 ratios within the range of 1.8 to 2.0 were deemed suitable for downstream applications.

High-quality RNA samples were reverse-transcribed into complementary DNA (cDNA) utilizing the First-Strand cDNA Synthesis Kit (OriGene, Heidelberg, Germany) in strict accordance with the manufacturer’s protocols [130]. The resulting cDNA was subsequently employed for quantitative PCR (qPCR) analysis to determine the expression levels of target genes, including PAR-1 and PAR-2. This approach enabled a precise assessment of OC’s impact on gene expression under inflammatory conditions, providing insights into its modulation of key signaling pathways.

### 4.13. Real-Time PCR for Quantification (qPCR)

Quantitative PCR (qPCR) was conducted with rigorous precision using the QuantStudio 5 Flex Real-Time PCR System (Applied Biosystems, Waltham, MA, USA; Thermo Fisher Scientific) to quantify the expression levels of PAR-1, PAR-2, TNF-α, and GAPDH in HT-29 and Caco-2 cells. SYBR Green chemistry was employed for its robust sensitivity and reliability in detecting double-stranded DNA during amplification, facilitating precise measurement of relative gene expression.

Primers for the target genes (PAR-1, PAR-2, TNF-α, and GAPDH) were meticulously designed using OriGene’s proprietary primer design algorithm, optimized for specificity, amplification efficiency, and minimal secondary structures. These primers underwent comprehensive in silico bioinformatics validation, including assessments of specificity against the human genome using BLAST (version BLAST+ 2.13.0) (Basic Local Alignment Search Tool). The results confirmed negligible off-target binding. Thermodynamic properties such as melting temperature (Tm), GC content, and dimerization potential were evaluated using Primer-BLAST and OligoAnalyzer, ensuring high amplification precision.

The quality of the primer sequences was further corroborated through their E-value and Bit Score metrics, as summarized in Table 1. The E-values of 0.0 for all primers unequivocally demonstrated perfect alignment with their intended target sequences, signifying the absence of random matches and unparalleled specificity. The Bit Scores, ranging from 2374 for GAPDH to 2861 for PAR-2, further confirmed the strength and precision of the primer–template alignments, with higher scores reflecting superior alignment quality. These metrics underscore the rigor of primer selection and validation, ensuring that all primers reliably amplified their respective targets with minimal risk of nonspecific amplification.

qPCR reactions were performed in technical replicates to ensure reproducibility, with no-template controls (NTCs) incorporated to detect potential contamination. The reaction conditions included an initial denaturation step at 95 °C, followed by 40 cycles of denaturation, annealing, and extension, with melt curve analysis performed to verify the specificity of amplified products. Relative gene expression levels were calculated using the 2^−ΔΔCt^ method, with GAPDH serving as the housekeeping gene.

To corroborate the quality and reliability of the qPCR data, amplification efficiency was determined using standard curves generated from serial dilutions of cDNA, with all efficiencies falling within the acceptable range of 90–110%. The high R^2^ values (≥0.99) of standard curves further validated the reliability of the assay.

This method provided an accurate quantification of changes in PAR-1, PAR-2, and TNF-α expression in response to OC treatment under inflammatory conditions. The integration of stringent primer validation, including robust E-value and Bit Score assessments, with optimized qPCR protocols ensured the reliability and robustness of the results, offering critical insights into OC’s modulation of inflammatory signaling pathways in CRC.

### 4.14. Calcium Signaling Assay

Intracellular calcium signaling, a fundamental regulator of cellular proliferation, apoptosis, and inflammation, is intricately linked to PAR-2 activation [131]. In CRC, PAR-2 activation engages phospholipase C (PLC), resulting in inositol-1,4,5-trisphosphate (InsP3)-mediated calcium release from intracellular stores. This calcium flux amplifies inflammatory pathways, including NF-κB signaling, which contributes to tumor progression [132].

To examine the modulatory effects of OC on calcium signaling in CRC models, HT-29 and Caco-2 cells were cultured to 70–80% confluence in 35 mm dishes containing 2 mL of complete growth medium. On the day of the experiment, the medium was aspirated, and cells were washed twice with pre-warmed phosphate-buffered saline (PBS) to eliminate any residual serum components that could interfere with dye uptake. A calcium-sensitive dye solution, Fluo-4, AM (2 µM; Thermo Fisher Scientific), was prepared in Hank’s Balanced Salt Solution (HBSS) supplemented with 1 mM CaCl_2_ and added to the dishes. Cells were incubated at 37 °C for 30 min, enabling intracellular esterases to hydrolyze the dye, followed by a further 30 min de-esterification at room temperature.

Post-dye loading, cells were exposed to LPS (10 µg/mL) to induce an inflammatory response. OC was subsequently administered at concentrations of 20, 50, 100, and 150 µg/mL, selected based on the existing literature highlighting its effective anti-inflammatory activity in comparable cell systems. After 24 h of OC treatment, live-cell fluorescence imaging was performed using a Leica DMi8 fluorescence microscope with excitation and emission filters specific to Fluo-4 (488 nm/516 nm). Fluorescence intensity was recorded in real time to quantify intracellular calcium levels under different experimental conditions.

This approach provided a precise assessment of calcium flux alterations in response to OC treatment in both control and LPS-induced inflammatory conditions in HT-29 and Caco-2 cells. The results are expected to reveal critical insights into the interplay between PAR-2 activity, calcium signaling, and the anti-inflammatory effects of OC in CRC.

### 4.15. Computational Modeling of PAR-2

The full-length amino acid sequence of PAR-2 was retrieved from the UniProt Knowledgebase [133] (Accession ID: P55085) [105]. The sequence, consisting of 397 amino acids, was formatted in FASTA format and used as input for AlphaFold v2.3, a state-of-the-art deep learning framework for high-accuracy protein structure prediction [134]. AlphaFold integrates multiple sequence alignment (MSA), deep residual neural networks, and physicochemical constraints to infer a three-dimensional structural model. The sequence was subjected to an MMseqs2-based homology search, where evolutionary homologs were identified from UniRef90, MGnify, and PDB70 databases to generate a statistically optimized MSA profile [135]. No experimentally determined homologous structures were explicitly used as templates to avoid biasing the prediction toward pre-existing crystallographic models.

The AlphaFold modeling pipeline was executed in monomer mode, generating five independent structural models ranked based on confidence metrics. These confidence metrics include the Predicted Local Distance Difference Test (pLDDT), Predicted Aligned Error (PAE), and interatomic Distance Difference Test (pIDDT) scores, each of which provides an independent validation of structural reliability. The model with the highest global pLDDT score was selected for further analysis.

The per-residue confidence of the predicted model was assessed using pLDDT, a continuous probability score ranging from 0 to 100, where values above 90 correspond to highly reliable atomic positioning, scores between 70 and 90 indicate moderate reliability, and scores below 50 suggest structural disorder or flexible regions. These pLDDT scores were extracted from the AlphaFold output files and mapped onto the structural model using Matplotlib (v2.3.0) for quantitative confidence visualization [134,136]. The model was further evaluated using PAE matrices, which quantify positional alignment uncertainty between residue pairs [38], with low PAE values (<5 Å) indicating rigid-body stability and high PAE values (>15 Å) denoting inter-domain flexibility. PAE heatmaps were generated to highlight regions of increased structural variability, particularly within the extracellular and intracellular loops.

To further validate the stability of the predicted structure, pIDDT scores were computed, which assess per-residue interatomic distance reliability independently of global structural constraints [137]. Residues exhibiting pIDDT > 80 were classified as structurally stable, whereas those with pIDDT < 60 were considered highly flexible or prone to dynamic conformational changes. The per-residue IDDT profile was plotted to enable a direct correlation between predicted structural disorder and functional loop dynamics.

Structural visualization and domain annotation were performed using ChimeraX v1.4 [138], a high-resolution molecular graphics software. The predicted model was rendered in cartoon representation, with extracellular loops, intracellular loops, and transmembrane helices distinctly highlighted. Structural color mapping was applied to distinguish helical, loop, and sheet conformations, but this visualization was not used for quantitative confidence assessment.

Figures representing different structural confidence metrics were generated to comprehensively assess the reliability of the predicted model. The sequence coverage plot was extracted from the MSA-derived output to illustrate homologous sequence conservation across the primary structure [139]. The pLDDT color-mapped structural model was plotted to visually delineate high-confidence transmembrane regions from low-confidence loop domains. The PAE matrix was represented as a heatmap, emphasizing areas of increased positional uncertainty, particularly in the extracellular and intracellular loops. Finally, the pIDDT per-residue plot was constructed to assess local structural stability and conformational flexibility across the predicted topology.

This comprehensive multi-metric validation approach enabled a systematic evaluation of PAR-2 structural reliability, allowing for the identification of rigid transmembrane domains and flexible loop regions, which are critical for understanding GPCR function and ligand-binding interactions.

### 4.16. Molecular Docking of OC with AlphaFold-Predicted PAR-2 Structure

The molecular docking of OC with PAR-2 was conducted using CB-Dock2 [140,141], a blind docking tool that integrates curvature-based cavity detection and AutoDock Vina-based molecular docking [142], with an additional homologous template-based refinement step. This computational pipeline allows for both structural cavity-based docking and template-guided ligand placement, enhancing binding site prediction accuracy. This study aimed to evaluate the potential binding interactions between OC and the transmembrane domain of PAR-2, considering its biological implications in colorectal cancer cells where PAR-2 is known to modulate inflammatory signaling.

The AlphaFold-predicted structure of PAR-2 was used as the docking receptor, selected based on its highest confidence pLDDT scores and structural stability metrics. Since PAR-2 is a transmembrane GPCR, the model was prepared by optimizing atomic positioning, maintaining transmembrane helical topology, and preserving extracellular and intracellular loop orientations. The molecular structure of OC was retrieved from the MedChem database, ensuring its lowest energy conformation and correct stereochemistry were maintained. The ligand was prepared in PDBQT format to define its torsional degrees of freedom for flexible docking.

CB-Dock2 employs a hierarchical workflow that begins with curvature-based binding pocket detection, where the query receptor structure is analyzed for potential ligand-accessible cavities based on geometric and electrostatic complementarity. The algorithm simultaneously retrieves protein–ligand complexes from its internal database to identify ligands with high topology similarity (FP2 ≥ 0.4). If a matching template ligand is identified, the sequence identity of the ligand-binding pocket is assessed, and templates with ≥40% sequence identity and pocket RMSD ≤ 4 Å are retained for template-based docking refinement. If no homologous template is available, the docking procedure proceeds using solely structure-based cavity detection.

Following binding site identification, molecular docking was performed using AutoDock Vina, which applies an empirical free energy scoring function to predict the optimal ligand conformation within the selected pocket. If a homologous template was available, the FitDock module within CB-Dock2 was utilized to align OC to the template ligand’s binding pose, refining dihedral angles and optimizing its initial placement before exploring conformational space. The docking simulations generated multiple binding poses, which were subsequently ranked based on binding energy (ΔG, kcal/mol) and pose confidence (PC-score). The final binding conformation was selected based on the lowest energy and highest confidence metrics.

The binding site interactions were analyzed using molecular visualization tools, including PyMOL and Discovery Studio, to map hydrogen bonding, hydrophobic contacts, and electrostatic interactions between OC and the transmembrane helical residues of PAR-2.

## 5. Conclusions

### 5.1. Limitations

Despite the promising mechanistic insights elucidated in this study, several methodological and conceptual limitations merit consideration.

*First*, the reductionist in vitro experimental paradigm employed herein, while providing valuable molecular mechanistic insights, does not fully recapitulate the intricate heterotypic cellular interactions within the TME. Immune cell infiltration, stromal remodeling, and microbiota-driven inflammatory modulation are crucial elements of CRC pathogenesis that cannot be fully captured in a two-dimensional model. These interactions include immune infiltrate composition, stromal–epithelial crosstalk, extracellular matrix remodeling, and microbiota-mediated inflammatory modulation. The absence of these factors may influence OC’s anti-inflammatory effects on PAR-2-driven oncogenic pathways. To address this limitation, future investigations should incorporate advanced three-dimensional biomimetic systems, including patient-derived organoids, heterotypic spheroid co-cultures incorporating stromal and immune components, and orthotopic xenograft models with humanized immune compartments [143,144,145]. These models would facilitate validation in a physiologically relevant context that preserves spatial architecture, intercellular communication, and paracrine signaling networks.

Second, this investigation was restricted to two colorectal adenocarcinoma cell lines (HT-29 and Caco-2), selected based on their differentiation status and constitutive PAR-2 expression profiles [34]. However, these models fail to capture the full molecular heterogeneity of clinical CRC, which encompasses distinct consensus molecular subtypes (CMS1-4) with divergent transcriptomic signatures, mutational landscapes, and immunological profiles [146,147,148]. CRC exhibits profound inter- and intra-tumoral heterogeneity, characterized by key oncogenic driver mutations, including microsatellite instability (MSI), KRAS^G12D/G13D^, BRAF^V600E^, PIK3CA^H1047R^, and TP53 alterations, which significantly modulate PAR-2 signaling, downstream inflammatory cascades, and therapeutic responsiveness [149]. Future studies should integrate a broader panel of genetically diverse CRC models, including HCT116 (MSI-high, KRAS^G13D^, PIK3CA^H1047R^), SW480 (APCmut, KRAS^G12V^, TP53mut), LS174T (MSI-high, KRAS^G12D^, PIK3CA^H1047R^), and patient-derived CRC cells spanning all four consensus molecular subtypes. Such an approach would enhance the generalizability of OC’s PAR-2-mediated anti-inflammatory effects across diverse CRC genomic backgrounds.

Third, our experimental design primarily investigated the acute molecular consequences of OC exposure on PAR-2 expression and inflammatory signal transduction. While this approach provides insight into immediate mechanistic alterations, it does not account for adaptive resistance mechanisms, compensatory pathway upregulation, or epigenetic reprogramming that may emerge under sustained therapeutic pressure [150]. Future longitudinal investigations should explore chronic OC treatment regimens to assess the potential for acquired resistance, including transcriptomic adaptations, epigenetic modifications, and feedback activation of compensatory inflammatory pathways. Notably, crosstalk between PAR-2 and other inflammatory GPCRs, such as prostaglandin receptors (EP1–EP4), chemokine receptors (CXCR1/2), or lysophosphatidic acid receptors (LPA1–LPA6), remains an open avenue of investigation [151,152,153]. Understanding these interactions will be essential in determining whether long-term OC exposure leads to compensatory reprogramming in inflammatory signal transduction.

Fourth, while our findings demonstrate OC’s selective downregulation of PAR-2 without perturbing PAR-1 expression, the potential modulatory effects on PAR-3 and PAR-4 remain unexplored. PAR-3 functions as an allosteric regulator of PAR-1/PAR-4 heterodimeric signaling complexes, while PAR-4 activation has been implicated in thrombin-mediated platelet aggregation and pro-metastatic phenotypes [21]. Both receptors exhibit context-dependent oncogenic or tumor-suppressive functions in CRC by modulating thrombin-induced ERK1/2 and p38 MAPK signaling cascades. Additionally, beyond the PAR family, this study did not assess whether OC affects other GPCRs that contribute to CRC pathophysiology, such as chemokine receptors (CXCR4, CXCR1/2) or prostaglandin receptors (EP1–EP4). While our findings support the specificity of OC’s effects on PAR-2, future investigations should incorporate broad GPCR profiling using transcriptomic and proteomic approaches to identify potential off-target interactions. Future investigations should incorporate comprehensive protease-activated receptor profiling, including PAR-3 and PAR-4 expression levels, activation dynamics, heterodimerization patterns, and downstream signaling interactions to determine whether OC exerts broader regulatory effects across the PAR family or demonstrates receptor subtype selectivity.

Finally, significant translational challenges exist regarding OC’s pharmacokinetic properties and bioavailability. Despite exhibiting potent in vitro anti-inflammatory and PAR-2 modulatory effects at low micromolar concentrations, OC’s systemic absorption, first-pass metabolism, plasma protein binding, blood–brain barrier penetration, and tissue distribution remain incompletely characterized [154]. OC contains reactive α,β-unsaturated aldehyde moieties, which render it susceptible to nucleophilic attack, potentially reducing its metabolic stability via rapid conjugation with glutathione or cysteine residues [155]. Future studies should focus on comprehensive in vivo pharmacokinetic profiling, quantitative structure–activity relationship (QSAR) analyses, and advanced drug delivery strategies. Strategies such as lipid-based nanoparticles, polyethylene glycol (PEG)-conjugated formulations, and nano-emulsified delivery platforms could enhance OC’s bioavailability, extend its circulatory half-life, and optimize tumor-specific accumulation through passive or active targeting mechanisms.

Collectively, these limitations highlight the need for preclinical validation in physiologically relevant models, expanded CRC subtypes, long-term resistance studies, broader PAR family investigations, and pharmacokinetic optimization to translate oleocanthal’s mechanistic potential into viable clinical applications.

### 5.2. Future Directions

Future studies should prioritize in vivo validation of these findings using animal models and patient-derived xenografts to assess the therapeutic efficacy of OC in a more clinically relevant setting. Advanced proteomics, particularly phosphoproteomics, could be employed to uncover post-translational modifications and signaling changes driven by OC [156]. Additionally, CRISPR-based approaches could be utilized to generate isogenic cell lines with PAR-2 knockout or overexpression to definitively establish causality and investigate off-target effects of OC [157]. Investigations into the synergistic potential of OC with conventional chemotherapeutic agents, such as 5-fluorouracil or topoisomerase inhibitors, could uncover combinatorial strategies to enhance CRC treatment [158]. Furthermore, advanced techniques like single-cell RNA sequencing could provide deeper insights into additional pathways modulated by OC, further elucidating its mechanism of action. The potential of OC to modulate other PAR family members or interact with the microbiota-associated inflammatory pathways also warrants exploration.

In conclusion, this study underscores the therapeutic potential of OC as a selective modulator of PAR-2-driven inflammation in CRC. As depicted in Figure 9, OC exerts its anti-inflammatory and tumor-suppressive effects primarily through the downregulation of PAR-2 expression, thereby disrupting key oncogenic and inflammatory signaling cascades integral to CRC progression. The suppression of PAR-2 inhibits TNF-α-mediated activation of the NF-κB pathway, leading to a significant reduction in pro-inflammatory cytokine production and downstream inflammatory transcriptional activation (*signaling axis 1*). This dampening of inflammatory signaling plays a crucial role in disrupting the tumor-promoting microenvironment in CRC. Additionally, by interfering with calcium flux and reducing intracellular calcium mobilization (Figure 9), OC may further attenuate the calcium-dependent activation of NFAT transcription factors, thereby limiting inflammation-induced tumorigenesis.

The impact of OC-mediated PAR-2 downregulation extends beyond inflammation, as it also exerts a significant effect on Wnt/β-catenin signaling, which is central to CRC proliferation, invasion, and chemoresistance (*signaling axis 2* in Figure 9). PAR-2 activation has been shown to stabilize β-catenin by inhibiting its proteasomal degradation, thereby sustaining oncogenic transcriptional programs that promote tumor growth, epithelial–mesenchymal transition (EMT), and stemness. One key mechanism through which this occurs is the recruitment of LRP6, a co-receptor in the Wnt signaling cascade, which stabilizes β-catenin by preventing its ubiquitination and proteasomal degradation. By disrupting this stabilization mechanism, OC indirectly promotes β-catenin degradation, thereby interfering with the transcription of oncogenic targets such as c-Myc and Cyclin D1. Given that the Wnt/β-catenin pathway is a primary driver of CRC progression and therapy resistance, this disruption highlights OC’s therapeutic potential as a modulator of key CRC signaling cascades.

Additionally, OC’s suppression of PAR-2 affects critical survival pathways, including the PI3K/Akt and MAPK cascades (Figure 9). These pathways are essential for maintaining cancer stem cell populations, promoting EMT, and facilitating metastatic dissemination. By attenuating PAR-2-mediated recruitment of LRP6 and Axin, OC disrupts the stabilization of β-catenin and inhibits downstream activation of Akt and ERK signaling. This inhibition not only suppresses tumor cell survival and proliferation but also enhances CRC cell susceptibility to apoptosis, as demonstrated in previous studies on OC’s pro-apoptotic effects. The ability of OC to downregulate these fundamental oncogenic pathways through PAR-2 modulation reinforces its potential as a multi-target therapeutic agent in CRC.

OC’s natural derivation from EVOO aligns with the benefits of the Mediterranean diet, positioning OC as a cost-effective, accessible, and safe option for CRC prevention and management. As depicted in Figure 9, OC’s broad mechanistic effects extend beyond PAR-2 downregulation, influencing multiple tumor-promoting pathways that are intricately connected within the CRC microenvironment. The figure provides a comprehensive overview of how OC interferes with inflammatory, survival, and metastatic signaling cascades, reinforcing its potential as a promising therapeutic strategy.

To bridge the gap between preclinical findings and clinical applications, future studies should focus on in vivo validation using patient-derived xenografts and CRC organoid models coupled with pharmacokinetic and bioavailability assessments to optimize OC’s therapeutic efficacy. Additionally, exploring OC’s synergistic potential with existing CRC therapies, such as immune checkpoint inhibitors and targeted therapies, may enhance its translational viability (Figure 9). Given the well-documented limitations of conventional CRC treatments, the inclusion of OC as an adjunctive or complementary therapy could offer an alternative approach to mitigate therapy resistance and improve patient outcomes. Clinical trials assessing OC-rich dietary interventions in high-risk CRC populations would provide essential insights into its preventive and therapeutic potential. As illustrated in Figure 9, OC’s ability to target multiple signaling pathways through PAR-2 downregulation highlights its broad spectrum of anti-cancer activities, positioning it as an attractive candidate for further translational research. However, further research is essential to translate these promising in vitro findings into clinically actionable therapies.

## Figures and Tables

**Table 1:** Oligonucleotide primers used for real-time quantitative PCR.

Gene	Primer Type	Sequence	Accession	E-Value	Bit Score
*GAPDH*	Forward (5′-3′) Reverse (5′-3′)	GTCTCCTCTGACTTCAACAGCG ACCACCCTGTTGCTGTAGCCAA	NM_002046	0.0	2374
*PAR-2*	Forward (5′-3′) Reverse (5′-3’)	CTCCTCTCTGTCATCTGGTTCC TGCACACTGAGGCAGGTCATGA	NM-005242	0.0	2861
*PAR-1*	Forward (5’-3’) Reverse (5’-3’)	GCTGTCCTACTGCTTGGAAGAC CTGCATCAGCACATACTCCTCC	NM_022002	0.0	2745
*TNF-α*	Forward (5’-3’) Reverse (5’-3’)	CTCTTCTGCCTGCTGCACTTTG ATGGGCTACAGGCTTGTCACTC	NM_000594	0.0	2449

*GAPDH*: glyceraldehyde-3-phosphate dehydrogenase; *PAR-2:* protease-activated receptor-2; *PAR-1:* protease-activated receptor-1; *TNF α:* tumor necrosis factor α.

## Data Availability

The datasets generated and/or analyzed during the current study are not publicly available but are accessible from the corresponding author upon reasonable request. Interested researchers may contact the corresponding author for data access inquiries, subject to compliance with any applicable privacy or confidentiality obligations.
